# Subcellular Enrichment Patterns of New Genes in *Drosophila* Evolution

**DOI:** 10.1093/molbev/msaf038

**Published:** 2025-02-07

**Authors:** Chuan Dong, Shengqian Xia, Li Zhang, Deanna Arsala, Chengchi Fang, Shengjun Tan, Andrew G Clark, Manyuan Long

**Affiliations:** State Key Laboratory of Subtropical Silviculture, Zhejiang A&F University, Hangzhou, Zhejiang, China; Department of Ecology and Evolution, The University of Chicago, Chicago, IL, USA; Department of Ecology and Evolution, The University of Chicago, Chicago, IL, USA; Department of Ecology and Evolution, The University of Chicago, Chicago, IL, USA; Department of Ecology and Evolution, The University of Chicago, Chicago, IL, USA; The Key Laboratory of Aquatic Biodiversity and Conservation of Chinese Academy of Sciences, Institute of Hydrobiology, Chinese Academy of Sciences, Wuhan, Hubei, China; Department of Ecology and Evolution, The University of Chicago, Chicago, IL, USA; Department of Molecular Biology and Genetics, Cornell University, Ithaca, NY, USA; Department of Ecology and Evolution, The University of Chicago, Chicago, IL, USA

**Keywords:** new genes, localization in subcellular compartments, localization patterns, gene evolution in subcellular compartments, protein–protein interaction networks

## Abstract

The evolutionary patterns of proteins within subcellular compartments underlie the innovation and diversification foundation of the living eukaryotic organism. The location of proteins in subcellular compartments promotes the formation of network interaction modules, which in turn reshape the architecture of higher-level protein–protein interaction networks. Here, we conducted the most up-to-date gene age dating of *Drosophila melanogaster* by employing recently available long-read sequencing genomes as references. We found that an elevated gene fixation in the most recent common ancestor of *Drosophila* genus predated the divergence between two *Drosophila* subgenera, and a significant tendency of these genes in *D. melanogaster* encode proteins that localize to the extracellular matrix, accompanying the adaptive radiation of *Drosophila* species. Proteins encoded by genes located in the extracellular space exhibit higher sequence divergence, suggesting a rapid evolutionary process. We also observed that proteins encoded by genes originating from the same evolutionary branches tend to co-localize in the same subcellular compartments, and proteins in the same subcellular compartment tend to interact with each other. The proteins encoded by genes that have persisted through deeper branches exhibit broader localization across multiple subcellular compartments, enhancing the likelihood of their integration into various protein or gene regulatory networks, thereby increasing functional diversity. These evolutionary patterns not only contribute to understanding the evolution of subcellular localization in proteins encoded by genes originating from different branches, but also provide insights into the evolution of protein–protein networks driven by the emergence of new genes.

## Background

Evolutionarily new genes are attracting increasing interest in understanding the evolution of gene functions and the genetic basis underlying biological diversity and phenotype innovation (Long et al. 2013). Such genes provide materials for studying fundamental molecular evolution patterns because they maintain distinct characteristics compared with old genes in terms of gene structure, epigenetic profiles, and transcriptional regulation (Werner et al. 2018). Genes can originate through more than a dozen distinct mechanisms (Chen et al. 2013). In addition to gene duplication, several other mechanisms contribute to the emergence of new genes (Zhang 2003; Long et al. 2013). For example, new genes can arise from noncoding DNA sequences and have been found to be prevalent in organisms such as *Oryza* (Zhang et al. 2019) and yeasts (Carvunis et al. 2012). New genes can perform important functions and are involved in diverse biological processes as shown in human disease analysis (Toll-Riera et al. 2009; Suenaga et al. 2014; Broeils et al. 2023), gametogenesis in *Drosophila* (VanKuren and Long 2018; Schroeder et al. 2020), early development in insects (Klomp et al. 2015; Kasinathan et al. 2020; Li et al. 2023), reproductive behavior (Dai et al. 2008), and brain evolution (Chen et al. 2012; An et al. 2023).

The development of new gene databases with accurate determination of gene ages for genomes of organisms is essential for further study of the evolution and molecular functions of new genes. Shao et al. (2019) expanded the online knowledgebase of gene ages, GenTree, to include species ranging from *D. melanogaster*, chicken, humans to a few other mammalian species. However, several limitations ought to be improved. First, the whole-genome shotgun sequencing used to generate short-read genomes cannot completely avoid uncertain or incorrect assemblies due to short repetitive sequences that may result in false positives in new gene identification (gene age dating accuracy depends on the quality of genome assembly). Second, the lack of the genomes from closely related outgroups of the *Drosophila* genus makes it difficult to identify new genes in the root branches and investigate the early evolution of new genes with the early *Drosophila* diversification. To advance molecular evolution research in *Drosophila*, it is crucial to develop an updated gene age database utilizing long-read genome sequences. Such a database will not only provide a more accurate understanding of gene origins and evolution but also facilitate the investigation of how new genes function and adapt within the context of subcellular compartmentalization. The subcellular compartments within the cells of organisms play fundamental roles in biological processes. Subcellular compartments further divide the components of the cell, which allows various biochemical reactions to occur in a compartmentalized and ordered manner, preventing intrinsic interference and enabling genes to function at specific times and locations (Thul et al. 2017). Consequently, investigating the evolution of new gene localization in subcellular compartments is valuable for understanding of gene function diversity and phenotype innovation. The evolution of proteins in terms of localization in subcellular compartments, encoded by genes that originated at different times (as measured in the phylogenetic branches), poses two intriguing questions. First, how does protein evolution occur in subcellular compartments after the birth of their corresponding genes? Second, how do distinct subcellular compartments influence protein–protein interactions? The *Drosophila* lineage (*Drosophila* subgenus lineage and *Sophophora* subgenus lineage) has diverged into roughly 1,600 species within the last ∼100 million years (O’Grady and DeSalle 2018), providing excellent material for studying new gene evolution and origination especially for the questions proposed the above.

To broaden our understanding of how evolution of proteins (encoded by genes with different origination time scales) regarding their localization in subcellular compartments, we included long-read sequencing genomes, including 10 long-read sequencing genomes, in our reference tree to infer gene age and comprehensively study these questions. In detail, the reference tree includes three long-read sequencing genomes on which our group had previously published (Flynn et al. 2020), *Drosophila virilis*, *Drosophila novamexicana*, *Drosophila hydei*, as well as five other long-read sequencing genomes (*Drosophila ananassae*, *Drosophila erecta*, *Drosophila persimilis*, *Drosophila azteca*, and *Drosophila orena*). The genomes of these species are from the *Drosophila* sequencing project PRJNA475270, which is hosted by three research teams. We also include the other two long-read sequencing genomes, *Scaptodrosophila lebanonensis* and *Bactrocera dorsalis* in our reference evolutionary tree, which are two evolutionarily related outgroup species to the *Drosophila* lineage. With the inclusion of *S*. *lebanonensis* and *B. dorsalis*, we can also investigate the gene birth at the most basal point of the *Drosophila* phylogeny which contains two subgenera, *Drosophila* and *Sophophora*. Briefly, we found an elevated gene fixation in the entire *Drosophila* genus, from the most recent common ancestor (MRCA) to the two major subgenera (*Sophophora* and *Drosophila*) even when introducing another species *Lissocephala sabroskyi* that has a close evolutionary relationship with *S. lebanonensis* (as shown in Figure 1 of Hopkins et al. (2024)). These new proteins, encoded by their corresponding genes, evolve to adapt to their specific cellular environments, shaped by their extracellular or intracellular localization, and exhibit distinctive evolutionary patterns. Additionally, newly born genes tend to be associated with extracellular localization, while proteins encoded by genes from deeper branches tend to localize in intracellular spaces. The co-localization tendency of proteins originating from the same branch promotes their likelihood of interacting with each other, elucidating the evolution of new genes within protein–protein interaction networks.

**Fig. 1.**
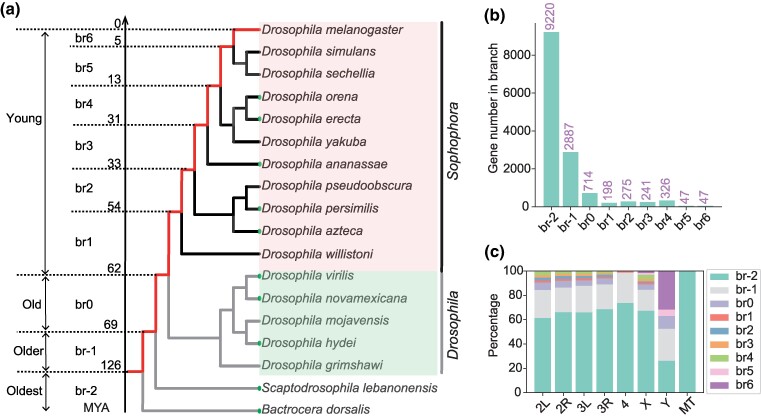
The tree employed for gene age dating of *D. melanogaster* and the gene distribution originating from different evolutionary branches. a) The evolutionary tree used for gene age dating of *D. melanogaster* is shown. Long-read sequencing genomes are represented by solid circles. Species belonging to the *Drosophila* subgenus and *Sophophora* subgenus are highlighted. All the genome links indicated in the tree are provided in supplementary table S1, Supplementary Material online, and the topology of the tree is provided in supplementary materials, Supplementary Material online. b) The bar plot shows the gene distribution across different branches from the oldest branch (br-2) to young branches (br1 to br6). The gene number is illustrated at the top of each bar. c) The distribution of genes originating from different branches among chromosomes is depicted, including autosomes, sex chromosomes, and the mitochondrial genome (MT). All the gene age data used for generating this figure are available in supplementary table S2, Supplementary Material online.

## Results

### Extensive Gene Fixation Was Detected Predating Subgenus Divergence, With a Higher Proportion of Protein-Coding Genes Linked to Sex Chromosomes

We utilized phylostratigraphy approach to date gene age. Within the framework of the phylostratigraphy approach, founder genes can be identified using either protein-based alignment (Domazet-Lošo et al. 2007) or genome-based alignment (Shao et al. 2019). In this study, we employed genome-based alignment to trace the emergence of founder genes. Our previous gene age annotations for *D. melanogaster* based on genome alignment relied on short-read sequencing genomes of reference species, without the inclusion of outgroup species of the *Drosophila* genus. To address this, we employed two closely related species, *Scaptodrosophila lebanonensis* (long-read assembly: GCA_003285725.2) and *Bactrocera dorsalis* (long-read assembly: GCA_030710565.1), as outgroups to investigate how many genes emerged in the early stages of *Drosophila* species evolution. Additionally, by introducing another eight long-read genomes (for a total of 10 long-read genomes), we obtained a higher number of detailed evolutionary branches on our phylogenetic tree (Fig. 1a and supplementary materials, Supplementary Material online). The genomes of the species in the tree can be downloaded via the links provided in supplementary table S1, Supplementary Material online. We categorized the gene age profiles of *D. melanogaster* into nine distinct evolutionary stages, denoting them from br-2 to br6 based on their originating time (Fig. 1a). Genes originating on branch-2 (br-2) represent the oldest genes (Oldest), genes on branch-1 (br-1) denote relatively old genes (Older), and genes on branch 0 (br0) are classified as old genes (Old) (the common ancestor of *Drosophila* species), and genes originating from branch 1 to branch 6 (from br1 to br6) are defined as new/young genes (Young) (Fig. 1a). Using computational pipeline based on parsimonious syntenic comparison for gene age classification (refer to our Methods section and the preprint (Fang et al. 2024) for more details), we identified 1,134 young protein-coding genes (genes from br1 to br6) in *D. melanogaster*, which accounts for approximately 8.1% (1,134/13,955) of all the dated protein-coding genes (Fig. 1b). Among the 1,134 young protein-coding genes, there are 198 protein-coding genes distributed on br1, 275 on br2, 241 on br3, 326 on br4, 47 on br5, and 47 on br6 (Fig. 1b and supplementary table S2, Supplementary Material online), respectively. The fixation rate refers to the genes that are retained after undergoing a series of complex events following their initial birth, which can be calculated by subtracting the number of gene deaths from the number of gene births, and then dividing by the time span. We estimate the fixation rate (the normalized number of branch-specific genes per million years) of young protein-coding genes to be approximately 18 genes per million years (1,134/62) which is roughly equivalent to the estimate in Shao et al. (2019) (1,070/62 = 17 genes per million years).

For the genes in br0 originated after the divergence between *S. lebanonensis* and the *Drosophila* genus, but before the divergence of the *Drosophila* and *Sophophora* subgenera. We identified 714 protein-coding genes in br0 by introducing genomes of *S. lebanonensis* and *B. dorsalis* (Fig. 1a and b). Branch br0 is estimated to approximately span 7 million years (MY) (69 to 62 MY), based on species divergence time (Fig. 1a). This result revealed a high gene fixation rate of 102 protein-coding genes per million years (714/7) emerging in the common ancestral *Drosophila* species over a 7-million-year period, far exceeding the 18 new genes per million years rate observed in the *D. melanogaster* lineage over 62 million years. We currently have only one species on br-1 (*S. lebanonensis*). Therefore, if gene loss (gene death) occurs frequently in the outgroup species (*S. lebanonensis*), it may lead to an overestimation of the number of gene fixations on br0, resulting in an inaccurate assessment of the gene fixation rate on that branch. To mitigate this overestimation of gene origination in br0 due to gene loss in br-1, we introduced a closely related species, *Lissocephala sabroskyi*. With this newly included species, we conducted a genome alignment between *D. melanogaster* and *L. sabroskyi*. We then checked whether the genes present in br0 also appear in *L. sabroskyi*. If these genes are found in *L. sabroskyi*, it indicates that the above assigned age may be erroneous due to gene loss in *S. lebanonensis*. Our analysis revealed that approximately 218 genes were lost. Consequently, the adjusted fixation rate for protein-coding genes on br0 is approximately 71 genes per million years ((714 − 218)/7), which also reflects an accelerated gene fixation rate compared with that in young branches (br1 to br6). Note that, due to our inclusion of an external species, *L. sabroskyi*, to estimate the fixation rate on br0, this estimation (71 protein-coding genes per million years) is closer to the true gene birth rate than the above estimation (102 protein-coding genes per million years).

Besides protein-coding genes, we also investigated whether there is a similar noncoding RNA (ncRNA) gene fixation pattern in the common ancestor branch of *Drosophila*. In the focal species of *D. melanogaster*, we obtained 604 young ncRNA genes representing ∼24.14% (604/2502) out of all annotated ncRNA genes (supplementary fig. S1a and table S3, Supplementary Material online). We estimated the young ncRNA gene fixation rate to be ∼10 genes per million years (604/62) after the divergence from the *Drosophila* subgenus. Among those young ncRNA genes there are 107, 275, 65, 139, 16, and 2 ncRNA genes distributed on br1 through br6, respectively (supplementary fig. S1a and table S3, Supplementary Material online). By introducing different long-read sequencing genome-based outgroup species genomes, 527 *Drosophila* genus-specific ncRNA genes were identified that predate the divergence of the two subgenera (supplementary fig. S1a, Supplementary Material online). This indicates a high fixation rate of ∼75 ncRNA genes per million years (527/7) in the MRCA, which is consistent with the result observed in the protein-coding genes. In summary, the fixation rate of genes was elevated before the divergence of the two subgenera, for both protein-coding and ncRNA genes.

Furthermore, our results revealed a chromosomal bias in the distribution of new protein-coding genes (br1 to br6), with the sex chromosomes (X and Y) exhibiting the highest proportion of new protein-coding genes compared with the autosomes (Fig. 1c). For example, there is a distribution of 11.1% new genes (br1 to br6) on the sex chromosome, while this proportion decreases to 7.5% on the autosomes (Chi-squared test, *P-*value = 3.3e-08). More specifically, the proportion of young protein-coding genes on the Y chromosome is the highest among all investigated chromosomes (Fig. 1c). This result indicates that the Y chromosome is also experiencing new gene birth events, which is contradictory to the conventional notion that Y chromosome evolves primarily through gene inactivation and loss. Using short-read genomic data, Tobler et al. (2017) showed that approximately 0.25 new genes/species/MY originate on the Y chromosome. Within br6, we identified six protein-coding genes in *D. melanogaster*, showing a higher fixation rate of new gene (6/5 = 1.2 genes/MY). This indicates that species-specific gene origination rate on Y chromosome can be higher than traditional thoughts (Tobler et al. 2017). However, this rate remains significantly lower than the overall new protein-coding gene origination rate (1.2 genes/MY vs. 18 genes/MY). The results mentioned above only consider the distribution of gene ages within chromosomes. When we examine the distribution of all new genes across different chromosomes, we find that the Y chromosome contains only seven young protein-coding genes, which accounts for a mere 0.6% (7/1137) of all young protein-coding genes. This suggests that gene birth on the Y chromosome is scarce compared with other chromosomes. Additionally, the Y chromosome is rich in repetitive sequences and sequencing gaps, making a comprehensive analysis of the entire Y chromosome essential for exploring the potential for new gene emergence, particularly using assembled genomes from long reads (Chang et al. 2022). Conversely, the overwhelming majority of protein-coding genes on chromosome 4 are the oldest genes (Fig. 1c). Furthermore, all the genes in the mitochondrial genome have the oldest protein-coding genes, suggesting that there is no protein-coding gene birth in this organelle (however, new protein-coding genes can be re-localized to the mitochondrion after gene birth [see Discussion section]) (Fig. 1c). Similarly, we also observed an enrichment of ncRNA on the sex chromosomes (with the exception of chromosome 4) and presented a similar chromosomal distribution trend compared with protein-coding genes (supplementary fig. S1b, Supplementary Material online).

We further employed 11 reference genomes (also including high-quality long-read sequencing genomes) to date the gene age of *D. melanogaster* (DroAge24) and compared the results with the GenTree database (supplementary tables S4 and S5, Supplementary Material online). The gene age of *D. melanogaster* listed in DroAge24 (supplementary table S4, Supplementary Material online) and gene age in GenTree (http://gentree.ioz.ac.cn/download/dm6_ver78_age.tsv) showed a high degree of consistency, with 94.3% of genes having the same estimated gene age. Additionally, a strong positive correlation in gene numbers across evolutionary branches was observed (*R*^2^ = 0.81, *P*-value = 4.3e-10), reinforcing the consistency. Genes with conflicting age were categorized as older (2.5%) or younger (3.2%) in DroAge24 compared with GenTree (supplementary table S5, Supplementary Material online). Furthermore, homologous data from FlyBase was used to further evaluate the genes with inconsistent ages mentioned above (2.5% older genes and 3.2% younger genes). The results showed that DroAge24 had more genes consistent with homologous-based ages compared with GenTree. In supplementary materials, Supplementary Material online, we provide a detailed comparison and explain the reason for further selecting 11 outgroup species as references. Notably, in our preprint article, we also evaluated the performance of gene age annotation pipeline using 11 sequenced genomes generated by NGS (Fang et al. 2024).

### The Extracellular Enrichment of Proteins Encoded by New Genes in *D. melanogaster*

**Fig. 2.**
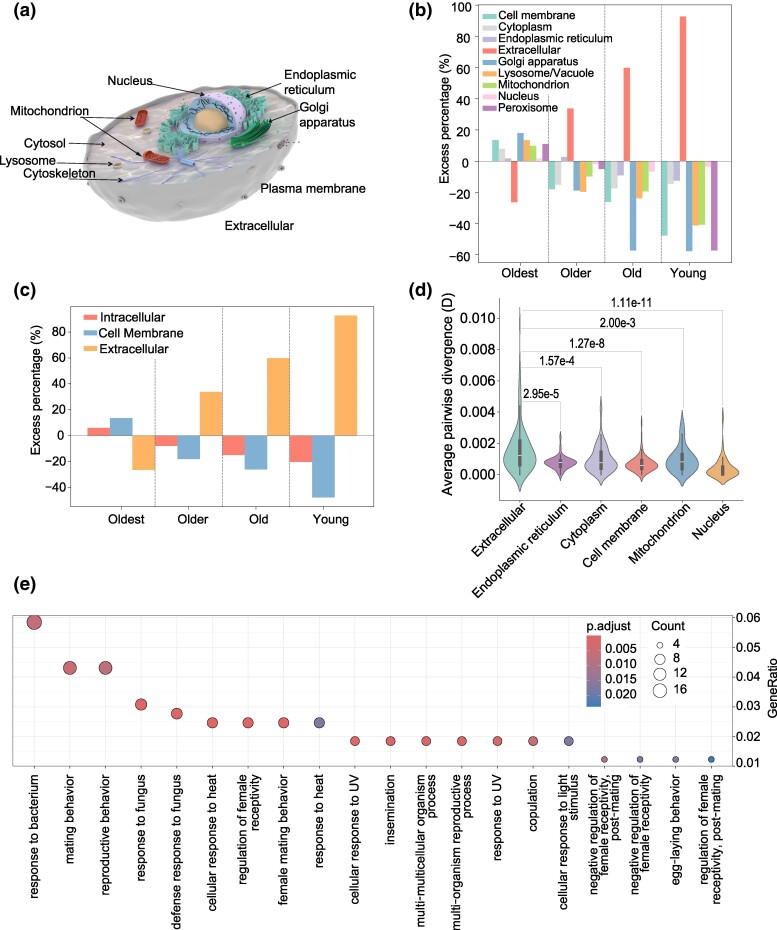
The enrichment pattern of subcellular compartments for proteins in *D. melanogaster*. a) The panel shows the major subcellular compartments of *D. melanogaster*. The subcellular compartments can be further divided into three parts including intracellular compartment, cell membrane, and extracellular compartment. b) The excess percentage was analyzed against gene age, comparing the oldest to young age group across nine subcellular compartments. c) The excess percentage was analyzed against gene age, comparing the oldest to the young branches across three subcellular compartments (intracellular compartment, cell membrane, and extracellular compartment). d) Sequence divergence was analyzed across different subcellular compartments. The *X*-axis represents the subcellular compartments and the *Y*-axis represents the average pairwise sequence divergence (*D* values). e) GO enrichment analysis was performed for young genes whose encoded proteins are exported into the extracellular compartment.

How has protein subcellular localization evolved during the process of gene birth and what is the potential role of subcellular localization evolution in *Drosophila*'s adaption? To explore these questions, we considered the nine major subcellular compartments of *D. melanogaster* (Fig. 2a). The subcellular compartments of proteins were predicted using DeepLoc2.0 local software (Thumuluri et al. 2022) with the accuracy prediction model activated by the option “-m Accuracy” (see Methods section for more details, supplementary table S6, Supplementary Material online). DeepLoc 2.0, using a pretrained protein language model, estimates the probability distribution of the query protein across ten subcellular compartments (one compartment is associated with plants; therefore, we excluded it from our analysis). According to the comparison between observed and expected localization numbers (see Methods section for more details), we found that new genes predominantly encode proteins that localize to the extracellular space (Fig. 2b) (Fisher exact test, *P*-value ≤ 0.01) (supplementary fig. S2, Supplementary Material online) and this was particularly the case for genes originating in branches br1, br2, br3, and br4 (supplementary fig. S3, Supplementary Material online). Starting from genes that originated in br0 (the common ancestor of *Drosophila*), proteins encoded by an elevated percentage of genes are significantly enriched in the extracellular space, compared with the entire background of genes (Chi-squared test, *P*-value = 3.3e-08) (Fig. 2b and supplementary fig. S2, Supplementary Material online). Additionally, our analysis at the intracellular, cell membrane, and extracellular matrix levels revealed that proteins in the extracellular matrix exhibited an initial accumulation during the early stages of protein production but showed a gradual decrease in abundance as gene birth events became more ancient (the enrichment percentage on br-2 and br-1 is lower than that on br0 and br1 to br6). In contrast, the pattern of intracellular and cell membrane localization exhibits the opposite tendency (Fig. 2c). Therefore, in *D. melanogaster*, different proteins encoded by genes on different branches appear to follow an evolutionary pattern where newly born genes are associated with extracellular localization, while those preserved in deeper branches tend to localize intracellularly (Fig. 2c). The proteins encoded by genes from the common ancestor period, as well as the subsequently generated proteins from new genes, exhibited an intensified extracellular enrichment phenomenon during the process of *Drosophila* adaptive radiation (Fig. 2b and c), which led us to hypothesize that proteins localized in the extracellular space may evolve rapidly to help *D. melanogaster* adapt to their environment, as these proteins frequently contact the external environment and are highly influenced by the environment.

To further test this hypothesis and investigate the evolutionary rate of these proteins, we utilized CD-HIT with parameters “-c 0.5 and -n 3” to cluster all proteins of *D. melanogast*er. Subsequently, proteins within the same cluster were aligned using MAFFT software (Nakamura et al. 2018) with default parameters. Our analysis revealed that proteins localized outside of cells exhibit significantly higher sequence divergence compared with clusters localized in other subcellular compartments (Mann–Whitney *U* test and the *P*-value < 0.05, Fig. 2d), implying their rapid evolutionary speed for the proteins localized in the extracellular space. This finding, together with the study from Julenius and Pedersen (2006), suggested that proteins (especially for the young protein-coding genes) located in the extracellular matrix evolve at a higher rate than those localized in other subcellular compartments. Furthermore, the research conducted by Smith et al. (2020) suggested a correlation between the extracellular matrix and phenotypic innovation, emphasizing the link between extracellular proteins and the phenotypic evolution of species. Considering that the evolution rate can be affected by gene age (Stein et al. 2018; Prabh and Rödelsperger 2022; Montañés et al. 2023), we constrained the clusters to the same or nearly same origination branch, then we examined the relationship between the average pairwise divergence and subcellular localization for protein clusters (supplementary fig. S4a, Supplementary Material online). Focusing on the clusters constrained to the same or nearly same evolutionary branches, our results show that protein clusters localized in the extracellular space exhibit a significantly faster evolutionary rate compared with those whose members are localized in other subcellular compartments, as determined by the Mann–Whitney *U* test (supplementary fig. S4b, Supplementary Material online). Meanwhile, we also noticed that proteins localized within the nucleus compartment exhibit the lowest sequence divergence, suggesting a relatively slow evolutionary speed for proteins within this organelle. This result satisfies our traditional view that important biochemical reactions usually take place inside the cell nucleus, which contains chromosomes and nuclear structural components. As a result, these proteins are subject to strong selective pressure. We are interested in the function of the new proteins encoded by gene localized in the extracellular space; therefore, we employed clusterProfiler to perform gene ontology analysis (Wu et al. 2021) for these young proteins localized in the extracellular space. It revealed that proteins encoded by new genes located in the extracellular matrix exhibit diverse functional enrichments, such as responses to heat, light, and resistance to fungi and bacteria (Fig. 2e). The photothermal response may enable *Drosophila* species to adapt to a wider range of temperature and altitude changes. Furthermore, we also observed that young proteins localized in the extracellular space also exhibited enrichment in reproductive behaviors such as mating and egg-laying (Fig. 2e). There are reports indicating that genes involved in reproductive behavior may be linked to the emergence of new species, further underscoring the significance of these proteins in biological evolution (Khallaf et al. 2020). In summary, our exploration into the evolution of subcellular protein localization provides a comprehensive understanding of the intricate dynamics between protein localization, and evolutionary processes during the evolution of *D. melanogaster*.

### Proteins Encoded by Genes Originating From the Same Evolutionary Branch Tend to be Co-localized in the Same Subcellular Compartments

**Fig. 3.**
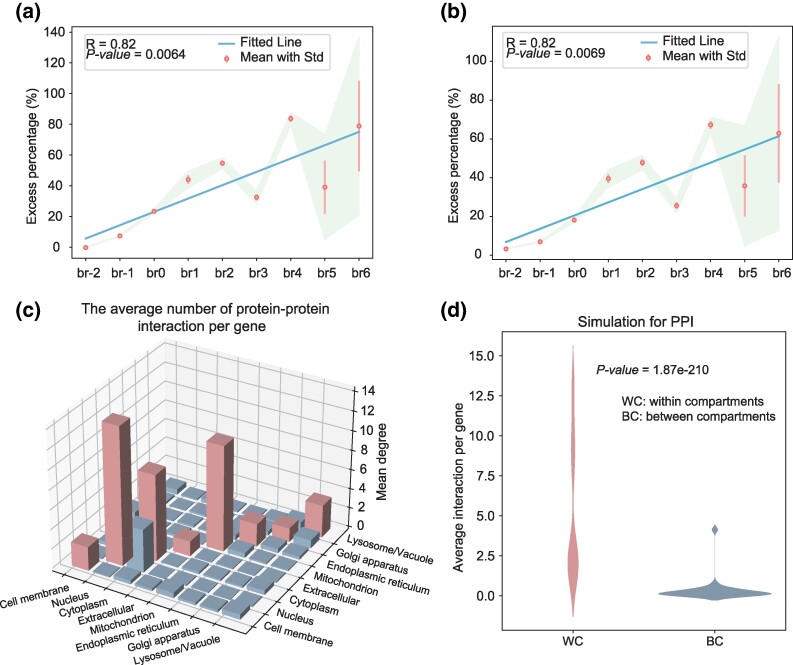
The figure compares co-localization analysis among protein pairs encoded by genes of different ages and includes a density analysis of protein–protein interactions localized in different subcellular compartments. a) Co-localizations were evaluated between protein pairs within the target branch and those formed by combining the target branch with other branches. b) Co-localization patterns were compared between the protein pairs within target branch and those protein pairs across all age groups. The solid lines in the graph represent the regression lines of co-localization tendency for protein pairs among different origination time, while the circle indicates the average of 100 comparisons, with standard deviation as error bars. c) The average number of protein–protein interactions per gene, both within and between subcellular compartments, is illustrated. The red bars on the diagonal represent protein interactions within the same subcellular compartment. d) The simulation result of protein–protein interactions within and between subcellular compartments are illustrated. The *P-*value indicated in (d) is measured based on the Mann–Whitney *U* test. WC means the average interaction per gene within subcellular compartments, and BC denotes the average interaction per gene between subcellular compartments.

In the above section, we observed that newly evolved genes (br1 to br6) are enriched in the extracellular matrix, while the proteins encoded by the oldest genes (br-2) tend to localize within the intracellular compartment, showing a different subcellular location pattern (Fig. 2b and c). This observation led us to speculate that protein pairs with similar origination branch may co-localize in the same subcellular compartment. To further decipher and test our speculation (the co-localization pattern of protein pairs encoded by genes originating from the same evolutionary branch), we performed a comparison of co-localization among proteins encoded by genes originating from intra- and inter-subcellular compartments (see Methods section for more details). We carried out a co-localization comparison for protein pairs solely within the target branch, as well as protein pairs arising from the combination of the target branch with other branches. In this comparison, we found that proteins with the same origination branch tend to co-localize in the same subcellular compartment (Fig. 3a). More specifically, this tendency is weakest in proteins encoded by the oldest genes (genes in br-2) (where the number of co-localizations within the oldest branch is almost equal to that between the oldest branch and other branches), which contrasts sharply with the young genes (br1 to br6) (Fig. 3a). For instance, in new genes, the proportion of protein pairs with the same origination branch that co-localize exceeds 30% compared to protein pairs between branches. Regression analysis indicates that the tendency of proteins to co-localize gradually weakens from the young branch to the oldest branch (Fig. 3a, *R* = 0.82, *P-*value = 0.0064). To further confirm this, we conducted another type of co-localization comparison between proteins originating from intra- and inter-originated times. This comparison strategy differs from the former one, as it primarily focuses on the proteins within the target branch and compares them with protein pairs across all age groups, rather than being limited to protein pairs solely between the target branch and other branches. Similarly, we have also discovered a consistent pattern (Fig. 3b, *R* = 0.82, *P-*value = 0.0069) compared with the former comparison (refer to Fig. 3a), which means that proteins originating from the same branch tend to co-localize within the same subcellular compartment, and this tendency is obvious in protein pairs encoded by young genes (br1 to br6), whereas it disappears in proteins encoded by the oldest genes. This observation may suggest that genes evolve new functions, enabling them to be sorted into various subcellular compartments to perform diverse functions. Therefore, the co-localization in the oldest gene group should be disrupted.

During a common origin period, these genes may experience similar selection pressures, leading to the proteins they encode exhibiting similar localization patterns within the cell. Therefore, we hypothesize that this may promote the involvement of these new genes with similar origination times in similar biological processes. Our observations indicate an intimate functional connection among new genes with similar origination branches, which seems to support the above hypothesis. To further validate the above speculation, we conducted KEGG pathway annotation via eggNOG-mapper (http://eggnog-mapper.embl.de/) (Cantalapiedra et al. 2021) (supplementary table S7, Supplementary Material online). Based on the age data and the KEGG pathway annotation, we compared the observed counts and expected counts of protein pairs in the same metabolic pathway. This comparison was conducted for a pair of proteins with the same origin branch, and we deemed that the pair of proteins having the same KEGG ID are co-functional, in that they participate in the same metabolic pathway. The expected participation in the same metabolic pathway for a pair of proteins can be estimated from the original distribution of the data, and this estimated frequency of co-function is 0.00604 (see Methods section for details). Accordingly, for the proteins having the same originate branch, the expected numbers of proteins co-functioned in the same KEGG pathway are 8,661,939 (the oldest branch, br-2), 121,333 (the older branch, br-1), 2,850 (the old branch, br0), and 2,746 (young branch), respectively. However, the observed co-KEGG pathway shows an excess of about 19.46%, 22.37%, and 37.2% compared with the expected numbers for br-1, br0, and young branches, respectively. The proteins in the oldest origin branch (br-2) exhibit an almost equal number of observed and expected co-participation in the same KEGG pathways (8,616,410 for expected and 8,661,939 for observed). The most ancient genes (genes in branch-2) do not show a co-participation phenomenon in metabolic pathways presenting the same trend with their co-localization in the subcellular compartment (proteins originating in br-2 do not exhibit co-localization within the same subcellular compartment). This can be explained from the perspective that the most ancient proteins have a long evolutionary history, and the phenomenon of co-involved gradually weakens and even disappears, obscuring the initial state after new gene birth. Therefore, we conclude that the proteins encoded by new genes with the same ages tend to engage in the same pathways (the same KEGG ID).

In summary, this observation suggests that the newly emerged proteins encoded by young genes often tend to co-localize within the same subcellular compartments and participate in the same biological processes (the same KEGG ID). However, this phenomenon may diminish or disappear in ancient genes.

### Proteins that Reside in the Same Subcellular Compartments Exhibit a Higher Propensity to Interact With Each Other

We observed the co-localized tendency for proteins with the similar originated branch. We next examined whether the subcellular localization patterns of proteins are associated with the formation of interaction networks, forming interaction zones in protein–protein interaction networks. To address this question, we calculated the average number of protein–protein interactions within the same subcellular compartments and compared them with the mean interactions for the proteins localized in distinct subcellular compartments. We found that the majority of physical protein–protein interactions consistently occurred between proteins localized within the same subcellular compartments, while proteins localized between subcellular compartments exhibited low-frequency physical interactions (Fig. 3c). This supports the notion that the formation of subcellular compartments shapes protein interaction modules to some extent. An exception is that protein pairs with frequent interactions are also found between proteins in the nucleus and cytoplasm (Fig. 3c), which means that lots of proteins still coordinate across different compartments. Additionally, we observed that the three main subcellular compartments for protein interactions are the nucleus, mitochondria, and cytoplasm. Proteins localized in these compartments have frequent interactions compared with proteins in other subcellular compartments. To further validate our observation, we conducted a study with 50 simulations. In each simulation, we randomly extracted 50,000 pairs of protein interactions from the entire dataset and divided them into two groups: interactions within compartments (WC) and interactions between compartments (BC). The simulated data demonstrated that the interaction frequency of proteins WC is significantly higher than the interaction frequency BC (Mann–Whitney *U* test, *P-*value = 1.87e-210) (Fig. 3d). We also noticed that proteins localized in the extracellular compartment have low protein–protein interaction frequency compared with the ones localized in other subcellular compartments (Fig. 3c). In summary, proteins residing in different subcellular compartments have a lower likelihood of interacting with each other, whereas proteins residing in the same subcellular compartments have intensive interactions (with the exception of extracellular compartment).

It was reported that the initial interaction of newly emerged genes at the periphery of the network, gradually integrated into the existing network over time (Zhang et al. 2015; Wei et al. 2016). Additionally, proteins encoded by genes with the same origin time tend to interact in the protein–protein interaction network (Capra et al. 2010). The evolutionary study of the subcellular localization of proteins encoded by new genes could explain the evolutionary patterns of new genes in protein–protein interaction networks. In the case of the *D. melanogaster*, new genes often locate in extracellular compartments, avoiding interaction with intracellular proteins, placing proteins encoded by new genes at the network's periphery. On the other hand, proteins encoded by genes with the same origin time tend to co-localization, predisposing them to preferential interactions in advanced protein interaction networks. Additionally, the result presented in this section can explain the fast evolution for proteins localized in the extracellular matrix. The lower frequency of interactions involving extracellular proteins may contribute to the relaxation of natural selection, thus can promote their fast evolution.

### Gene Age-Related Broadening of Subcellular Protein Localization Patterns and Changes in Subcellular Localization May Promote the Innovation of Protein Functions

**Fig. 4.**
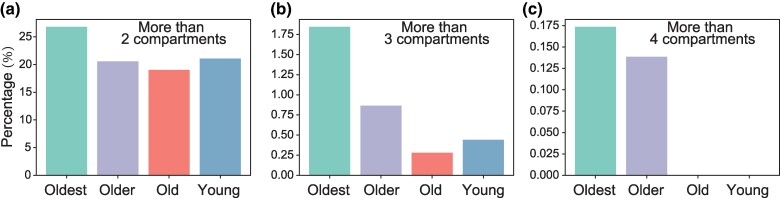
The proportion of proteins encoded by genes that are localized to more than a certain number of subcellular compartments. The *X*-axis represents gene categories (young [br1 to br6], old [br0], older [br-1], and oldest [br-2]). The *Y*-axis represents proportion.

Given the functional diversity of old genes and the limited functions of new genes, we hypothesize that the proteins encoded by the oldest genes (br-2) may exhibit more diverse subcellular localization than those encoded by new genes (br1 to br6). To test this, we measured the percentage of genes with proteins localizing to several subcellular compartments across four gene age catalogs (oldest, older, old, and young, which correspond to branches br-2, br-1, br0, and br1 to br6) in *D. melanogaster*. We observed that 26% of proteins encoded by br-2 genes localized to more than two subcellular compartments and that the proportion of proteins located in more than two subcellular compartments decreased in the young branch (Fig. 4a). Based on the same statistical pipeline, we calculated the proportion of genes whose proteins localized to more than three and four subcellular compartments in each age class (br-2, br-1, br0, and br1 to br6), respectively. We found that proteins encoded by oldest genes tend to localize to a wider range of compartments than proteins encoded by relative younger genes (Fig. 4b and c). The observed percentage of proteins encoded by both young and old genes, which are localized to more than four compartments, decreases to zero, shaping a comparison with protein encoded by gene on br-2 (oldest) and br-1 (older) (Fig. 4c). Together, these results support a conclusion that the proteins encoded by the oldest genes exhibit a broader range of subcellular localization compared to those encoded by young genes.

**Fig. 5.**
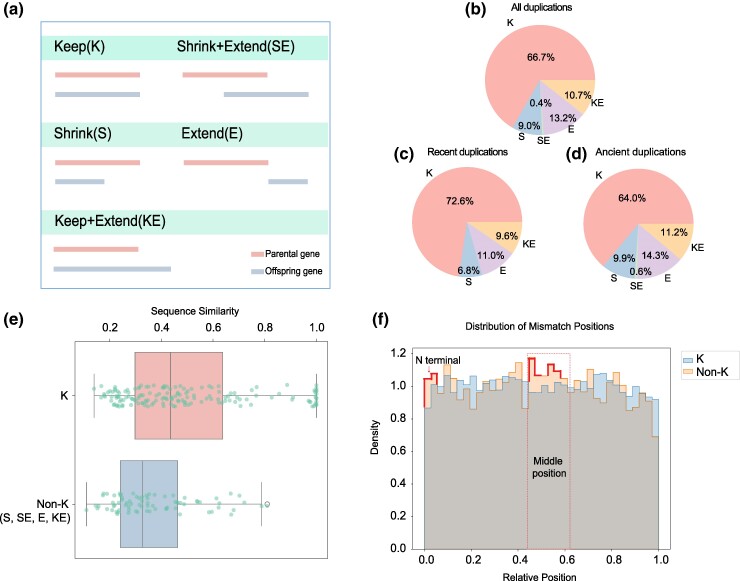
The subcellular localization divergence of proteins encoded by paralogous gene pairs in *D. melanogaster*. We define duplication events as an ancient duplication if the parental gene is located in the br-2 and the offspring gene is located in the br-1 or br0 branch. Additionally, if the parental gene is located in the br-1 and the offspring gene is located in the br0, they are also considered ancient duplications. a) Patterns of subcellular localization changes between parental genes and their offspring genes. The first bold line under each category (K, SE, S, E and KE) represents the parental genes, and the second bold line under each category represents the offspring genes. The length of the line represents the range of subcellular localization, with the longer line indicating a relatively broader localization range. The overlapping sections of two lines represent the overlap in subcellular localization between proteins encoded by the parental and offspring genes. b) The percentage distribution among the five localization patterns for all paralogous gene pairs. c) The percentage distribution among the five localization patterns for recent duplication gene pairs. d) The percentage distribution among the five localization patterns for ancient duplication gene pairs. e) The comparisons of the global sequence similarities for gene pairs between K and non-K groups. f) The comparison of mutated positions between the K and non-K groups.

We categorize the paralogous gene pairs into two types: ancient duplication and recent duplication (see Methods for this classification). Then, based on the branch of the paralogous gene pairs, we further defined the patterns of subcellular localization changes of proteins encoded by new (offspring) genes relative to their corresponding parent genes, which can be classified into five catalogs (K, SE, S, E, and KE) (Fig. 5a) (see Methods section). From an overall perspective with both ancient and recent duplication, our results show that approximately 66.7% of parent and new genes maintain the same subcellular localization (K) (Fig. 5b). In other words, 33.3% of gene pairs exhibit at least one distinct subcellular compartment localization pattern (SE, S, E, and KE), which aligns with a previous estimation in *Saccharomyces cerevisiae* (Marques et al. 2008). When we specifically examine recent duplication, we find that the proportion of genes maintaining identical subcellular localization is around 72.6%, and about 27.4% of parent and new (offspring) genes exhibit differences in subcellular localization (Fig. 5c). As the divergence time increases between parent genes and offspring genes, the pattern of differences in localization has expanded, resulting in a drop to 64% in the percentage of genes that maintain identical subcellular compartments (Fig. 5d). This analysis reveals that 27.4% (for recent duplications) to 36% (for ancient duplications) of duplicate gene pairs localize to at least one distinct subcellular compartment. In summary, our analysis in this section demonstrates that both recent and ancient duplicate genes may have evolved new functional roles through changes in their subcellular localization.

Based on the above analysis, we hypothesize that gene pairs (new genes and their parental genes) in the K category have relatively fewer sequence changes compared to gene pairs in other categories (KE, E, SE, and S). To validate this hypothesis, we perform global pairwise sequence alignments for paralogous gene pairs in the K category and those in other categories using MUSCLE. Our result showed that the gene pairs in the K category exhibit greater sequence similarity, while gene pairs in non-K categories (KE, E, SE, and S) showed weaker sequence similarity (Fig. 5e). The sequence similarity in the K category was significantly greater than that in the non-K categories (Mann–Whitney *U* rank test with parameter alternative=“greater”, *P-*value = 0.00037) (Fig. 5e). This suggests that sequence changes contribute to the divergence of subcellular location between parent and offspring gene pairs. Furthermore, we explored the mutation difference in the K and non-K gene pairs. To do this, we used MUSCLE to align the paralogous gene pairs in both catalogs (K and non-K). We then calculated the relative mutation position by dividing the actual mutation position by the total alignment length. Based on the mutation position, we compared the differences between K and non-K gene pairs. Our result showed that most of the mutations in the non-K group occur in the N-terminal and middle position (Fig. 5f). Mutations at the N-terminal can easily affect the signal peptide, and as a result, such mutations may alter the protein's localization, which is consistent with our observation. The comparison revealed a significant difference in the mutation position between gene pairs in the K and non-K groups (Mann–Whitney *U* test, *P-*value = 0.0094), especially for N-terminal and middle position. In summary, this suggests that mutations in the N-terminal and middle regions of the protein may drive the evolution of subcellular localization, leading to functional divergence between homologous proteins.

## Discussion

### Long-Read Sequencing Genomes and Gene Age Dating Pipeline Improve New Gene Annotation

Our dating pipeline introduced several improvements (Fang et al. 2024). First, the pipeline addresses the impact of repetitive sequences on whole-genome alignment by uniformly handling these sequences. Specifically, we unmasked the sequences in published genomes and then re-masked all sequences using a consistent method, thereby avoiding system errors caused by different masking strategies. Second, the pipeline processed the masked exon sequences in the target species. Based on the annotation files of the target species, we unmasked the exon regions to avoid alignment errors and gene age mis-assignments caused by the exclusion of exon regions from the alignment. Refer to our preprint paper for more details (Fang et al. 2024). Additionally, the incorporation of long-read sequencing reference genomes can reduce sequencing gaps and minimize the gene age dating errors. Our analysis results indicate that the annotation pipeline and the incorporation of long-read sequencing genomes provide a more accurate gene age list compared with GenTree, as supported by consistency analyses, expression studies, and homologous data validation from FlyBase (supplementary materials, Supplementary Material online). For rapidly evolving genes that frequently undergo duplication and loss, inferences about gene age may be inaccurate. However, these challenges can be mitigated through several measures. First, enhancing the quality of genome sequencing for outgroup species and reducing sequencing gaps is essential. Second, incorporating multiple species within the same outgroup branch can help avoid errors in gene age annotation due to gene loss. Additionally, including more closely related species as outgroups may further prevent annotation errors, as fewer genes are likely to be lost over a shorter evolutionary time span.

### The MRCA of the Two *Drosophila* Subgenera Exhibited an Elevated Number of Gene Fixations: Evidence From the Gene Age Dating of *Drosophila virilis*

We demonstrated that the MRCA of the *Drosophila* subgenera acquired and retained an elevated number of genes based on the gene age analysis of *D. melanogaster*. To further support this finding, we also identified new genes in *D. virilis* by adding more species, resulting in a phylogeny with 25 species and 11 branches (from br-2 to br8) (supplementary fig. S5a and supplementary materials, Supplementary Material online). We identified 679 MRCA-specific protein-coding genes that appeared in the MRCA (supplementary fig. S5b and table S8, Supplementary Material online). The protein-coding fixation rate in this branch is 97 protein-coding genes per million years (679/7) which is much higher than the average fixation rate of 11 genes per million years (701/62) in young branches (br1 to br8). Thus, we revealed that an elevated gene fixation occurred in the specific evolutionary branch, just preceding the subgenus divergence between the *Drosophila* and *Sophophora* lineages. Additionally, we also observed a similar phenomenon based on the age distribution of the ncRNA genes (supplementary fig. S5c and table S9, Supplementary Material online). In summary, the ancestral branch of the MRCA exhibits an elevated gene fixation, which is supported by the gene age distribution of *D. melanogaster* and *D. virilis*. This implies that the MRCA underwent extensive changes in its protein-coding and ncRNA genes during its speciation and subsequent evolution in a short period, likely leading to the diversification of *Drosophila* species as seen today (O’Grady and DeSalle 2018). In our calculation of the fixation rate on br0, we considered closely related species. For example, the divergence time between species on branch br0 and those on br-1 is approximately 7 million years (69 to 62). Over such a relatively short period, the number of gene losses is expected to be lower.

### The Subcellular Localization Evolution of Protein-Coding Genes Promotes Functional Novelties

By comparing the subcellular localization of orthologous genes in different lineages, people can preliminarily explore whether the gene's function is shifting as it evolves. For instance, if the same gene is localized to the nucleus in one lineage but to the cytoplasm in another, it is that this shift in localization may reflect a functional divergence between the species. This could imply that selective pressures in different lineages have driven the gene to adapt its function to different cellular environments. This difference of subcellular location contributes to their functional divergence in the regulation of pluripotency (Guo et al. 2020). Another example is the Rab family proteins (Stenmark and Olkkonen 2001). Rab proteins play a crucial role in vesicle formation, transport by associating with different membrane systems. Different Rab members are localized to distinct organelles and membrane systems, ensuring efficient transport of materials along specific pathways within the cell. This example illustrates the change of subcellular localization can influence functional divergence. Based on the above examples, it can be inferred that multi-subcellular localizations can promote the diversification of biological functions of genes.

The subcellular localization of proteins is influenced by a variety of factors. For instance, the nuclear localization signal (NLS) that facilitates the translocation of proteins from the cytoplasm to the nucleus (Lu et al. 2021). Conversely, the nuclear export signal (NES) is a short peptide sequence that mediates the export of proteins from the nucleus to the cytoplasm (Kutay and Güttinger 2005). Additionally, the transmembrane domain (TMD) is characterized by a hydrophobic amino acid sequence, enabling proteins to traverse cell membranes (Fink et al. 2012). The signal peptide (von Heijne 1990), typically located at the N-terminus, directs newly synthesized proteins to the endoplasmic reticulum (ER). The mitochondrial transit peptide (MTP) guides proteins from the cytoplasm into the mitochondria. In summary, the amino acid change in those position can change a protein's subcellular location. Based on the above facts, there are many factors that can lead to changes in protein subcellular localization. Sometimes, it is caused by mutations at the N-terminus, or by mutations in certain amino acids in the middle, or other changes in subcellular localization signals. In more complex cases, a combination of multiple mutations may lead to changes in protein subcellular localization.

In our work, we observed that the number of subcellular compartments they localize to become broader from the young branch to the oldest branch. According to the above discussion, modifying just a few specific amino acids can alter the subcellular localization of a protein. The subcellular locations of proteins encoded by genes that affect functional diversity can be discussed from several aspects. One possibility may be that as genes acquire more variable splicing forms as genes become older (Su et al. 2006), producing more encoded proteins, each carrying different subcellular localization signals to guide their localization with different subcellular compartments; Secondly, proteins encoded by genes are targeted to different subcellular compartments based on sorting mechanisms and localization signals within the cell, enabling them to function independently within their designated spaces. Different subcellular compartments may have distinct physicochemical properties, which establish diverse subcellular environments unique to each subcellular compartment. This compartmentalization directly impacts the modular organization of protein interactions or regulatory networks, which can lead to differences in the construction of cellular or tissue functions. Third, different subcellular compartments exert different selection pressures on their respective proteins and thus promote functional diversity because different subcellular compartments possess varying physicochemical properties. On the other hand, proteins within the same subcellular compartments tend to interact frequently with a lower frequency of protein interactions across subcellular compartments. Obviously, if a protein encoded by a gene can localize to multiple subcellular compartments, the protein encoded by this gene will participate in protein interaction networks across various subcellular compartments, thereby promoting functional diversity. Although subcellular compartments segregate the protein–protein interaction module of different proteins to some degree, lots of proteins still coordinate across different compartments. For example, we observed the proteins residing in the nucleus and cytoplasm also frequently interact (Fig. 3c).

Our data suggest young genes typically evolve faster than older genes, as they are less constrained due to the fact that their proteins are localized in extracellular spaces (Fig. 2b to d) and exhibit relatively fewer physical protein–protein interactions (Fig. 3c). This implies that proteins encoded by young genes might evolve the properties of being re-localized to other subcellular compartments in a relatively short time. We used mitochondrion as a case study to explore and discuss this because the mitochondrion is an ancient eukaryotic organelle (Sagan 1967). Based on the gene annotation of *D. melanogaster* (Drosophila_melanogaster.BDGP6.32.57.gtf) and the subcellular compartment information, we observed a progressive enrichment pattern for mitochondrial localization from the young genes to the oldest gene (Fig. 2b). However, we also found that 54 young genes (br1 to br6) encode proteins that can localize to mitochondria after their emergence, accounting for approximately 4% (80/1994) (the 54 young genes encode a total of 80 proteins and 1,994 proteins are localized in the mitochondria) of the total mitochondrial protein count. This result suggests that newly emerging genes can also contribute to mitochondrial functional innovation and its evolution, probably playing a role in the subsequent improvement of mitochondrial functions. To test the function of these proteins, we annotated the function of these genes and found that the functions of these genes are mainly enriched in the structural components of mitochondria, such as mitochondrial envelope and mitochondrial membrane (supplementary fig. S6, Supplementary Material online). The rates and mechanisms by which protein-coding genes evolve additional signals allowing them re-localize to multiple subcellular compartments remains to be explored and may be essential to understanding the functional diversity of proteins.

## Methods

### Methodology for Gene Age Determination

In general, the age of a gene can be determined by its distribution in a group of closely related species in a parsimonious framework to infer for new genes (Long et al. 2013). At the genomic level, a synteny-based approach is shown to be powerful to obtain orthologous information for genes between species to identify new genes that originated recently because of the conserved property of synteny in the short time-scale of *Drosophila* genus. The pipeline and algorithm have been created and applied to date the gene ages in the *Drosophila* genomes as the Zhang2010 (Zhang et al. 2010) and the GenTree (Shao et al. 2019), revealing various evolutionary patterns and functional roles. They or in slightly different computational platforms were also used widely to study new gene evolution in other organisms, ranging from mammals to flowering plants (Zhang et al. 2011; Jin et al. 2021). To improve computational speed, consistency and accuracy in gene age determination, a third-generation gene age dating tool, called GageTracker (the Gene Age Tracker) (it is freely accessible at https://github.com/RiversDong/GageTracker), was created recently and applied to *Drosophila* genomes (Fang et al. 2024. bioRxiv. https://www.biorxiv.org/content/10.1101/2024.08.28.610050v1). A technical advance was achieved by examining both micro-synteny within a gene and macro-synteny among genes at a chromosomal scale when searching for high-quality genome alignments necessary for the determination of orthologous relationships of genes. This approach is well facilitated with the third-generation sequencing data such as long-read sequencing genomes in this study. Compared with the earlier pipeline utilized in the GenTree database, the new algorithms and pipelines in the GageTracker significantly improved the speed, consistency, and accuracy in age determination in *Drosophila* (Fang et al. 2024). To understand the evolution of subcellular compartments brought by new genes, we generated gene age data for species in the lineages of two subgenera of *Drosophila*. All the relevant procedures are given below.

### Outgroup Species Used for the Phylogenetic Tree

To date the gene ages of *D. melanogaster*, we obtained the topology of phylogenetic trees containing the reference species based on the TimeTree database and our previous work (Zhang et al. 2010; Kumar et al. 2022) (supplementary materials, Supplementary Material online). In total, we used 17 outgroup species for gene age dating of *D. melanogaster* resulting in nine branches ranging from branch-2 (br-2) to branch 6 (br6) (Fig. 1a). We further divided the nine age branches of *D. melanogaster* genes into four categories. Genes in br-2 indicate that these genes originated at least br-2. Therefore, we consider br-2 to be the oldest origin branch in the current age classification of all genes. The br-1 denotes the older group, br0 is referred to as the old group, and genes originating from br1 to br6 are defined as new/young genes in this study. All of the genomes used in this work can be downloaded via the links provided in supplementary table S1, Supplementary Material online, which also includes the reference genomes used for gene age dating of *D. virilis*.

### Whole-Genome-Based Alignment and Gene Age Inference

The traditional way of identifying new genes is based on comparing the identity of gene sequences alignment and exon–intron structures. As gene duplication is a primary source of gene birth (Zhang et al. 2010), new genes derived from duplication would have similar sequences in reference species due to the sequence similarity between ancestral and duplicated genes. DNA duplication can lead to “many-to-one” or “many-to-many” orthologs in other species and are often treated as ancient genes when using traditional methods to infer gene age. Our group developed a synteny-based method to solve this problem (Zhang et al. 2010). The method utilizes whole-genome alignments to detect whether there is a syntenic ortholog in outgroup species for query genes (this pipeline can be available at GitHub, https://github.com/Zhanglab-IOZ/SBP_age-dating). We incorporated several enhancements to the age dating pipeline GageTracker (the source codes are available at https://github.com/RiversDong/GageTracker and its methodology article (Fang et al. 2024)). In this work, the tool GageTracker was utilized for age inference. Basically, this tool addressed the potential influence of repeat and sample sequences present in reference and query genomes (the focal species designated as the query/focus and the outgroup as the reference), which could impact the quality of genome alignment and subsequently affect gene age inference. Additionally, the use of masked reference genomes from different research groups may introduce technological bias. To mitigate these issues, we first unmasked all reference and focal genomes and then uniformly masked focal genomes again using WindowMasker and TANTAN (Morgulis et al. 2006; Frith 2011). Secondly, to prevent the masked exon regions in genes from hindering the alignment of query-subject genome pairs of gene regions, we unmasked any exon regions in the genome that were masked by WindowMasker and TANTAN before genome alignment. The exon regions were identified according to the gene annotation downloaded from ENSEMBL (Drosophila_melanogaster.BDGP6.32.57.gtf). Note that GageTracker integrates the LAST program (version 1282) (Kiełbasa et al. 2011) (https://gitlab.com/mcfrith/last/-/tree/1282?ref_type=tags) for genome alignment with parameters “-C2 -u0 -m50 -p HOXD70”. GageTracker also structured genome alignments between the focal and reference genomes into syntenic nets using chaining and netting procedures, following the guidelines outlined at UCSC Genome Browser http://genomewiki.ucsc.edu/index.php/Whole_genome_alignment_howto. This process yielded comprehensive whole-genome alignments. The CTL files used by GageTracker are available in supplementary materials, Supplementary Material online.

To minimize the risk of false orthologous alignment syntenic nets, GageTracker computes the reciprocal best syntenic nets, following the instructions provided at UCSC Genome Browser website http://genomewiki.ucsc.edu/index.php/HowTo:_Syntenic_Net_or_Reciprocal_Best. Utilizing the masked regions identified by WindowMasker, TANTAN, and the annotations of focal species *D. melanogaster* (Drosophila_melanogaster.BDGP6.32.57.gtf), GageTracker also calculates the proportion of repetitive and simple sequences within all exons of a gene (the proportion of masked exon sequences within all exons of a gene). Genes were excluded in our current analysis if this percentage exceeded 70%. The gene annotation of *D. melanogaster* was downloaded at the ENSEMBL database (https://ftp.ensembl.org/pub/release-110/gtf/drosophila_melanogaster/Drosophila_melanogaster.BDGP6.32.57.gtf.gz). The download links of the other reference genomes can be available at supplementary table S1, Supplementary Material online.

Besides dating the ages of protein-coding genes, we also annotated the ages of ncRNA genes in *D. melanogaster*. Note that the ncRNA genes presented here refer only to those listed in the annotation file Drosophila_melanogaster.BDGP6.32.57.gtf. The assembly version GCF_003285735.1 of the *D. virilis* genome did not reach the chromosomal level, but it provides gene annotations. Although the assembly version GCA_007989325.2 reached the chromosomal level, its annotations are not available from NCBI. Therefore, when annotating the gene age of *D. virilis*, we used version GCF_003285735.1. However, when annotating the gene age of *D. melanogaster*, we used version GCA_007989325.2 of *D. virilis* as the reference genome.

### Detecting Protein Subcellular Localization

DeepLoc2.0 is a widely used tool to perform the prediction of protein subcellular localization, which achieves state-of-the-art performance by including pretrained protein language models, ProtT5 (the ProtT5 model contains 2.1 billion protein sequences) (Elnaggar et al. 2021) and ESM1b (Rao et al. 2020). Additionally, DeepLoc 2.0 can distinguish between nine signal types with high accuracy in most cases (Thumuluri et al. 2022). We utilized DeepLoc 2.0 to predict the subcellular locations of *D. melanogaster*'s proteins (Drosophila_melanogaster.BDGP6.32.pep.all.fa). If the probability of a query protein being localized to a certain subcellular compartment is greater than 0.5, it can be considered as localized to that compartment. Therefore, a protein can be assigned to multiple subcellular compartments in the prediction results. To accurately detect the proteins’ subcellular compartments, we activated the accuracy model with the parameter “-m Accurate”. The prediction framework of this model will automatically load the pretrained protein language model, ProtT5-XL-BFD (Elnaggar et al. 2021), which can be available at the link of https://huggingface.co/Rostlab/prot_t5_xl_uniref50. The prediction results included ten subcellular compartments. However, since plastids are plant-specific organelles and not relevant to our current analysis, we excluded this organelle from our investigation. All prediction results can be available in supplementary table S6, Supplementary Material online. Note that some protein IDs in Drosophila_melanogaster.BDGP6.32.pep.all.fa are not present in the annotation file Drosophila_melanogaster.BDGP6.32.57.gtf. To quantify subcellular localization preference for proteins encoded by genes of a specific evolutionary branch (or age group), we compared the observed number of proteins localized to specific compartments with the expected number under random localization. For a given branch, we first calculated the proportion of genes assigned to that branch relative to the total number of genes. This proportion was then used to determine the expected distribution of proteins across subcellular compartments under random localization. The expected count of proteins localized to each subcellular compartment within a branch (or age group) was calculated based on these proportions. Finally, we assessed the enrichment level of subcellular localization by comparing the observed counts with the expected counts for each subcellular compartment under each age branch (or age group).

### The Evaluation of Co-localization for Proteins Encoded by New Genes

To measure the co-localization for proteins encoded by new genes within the same age group, we first calculated the different combinations of protein pairs within each age group, denoted as Intra_num. Subsequently, we counted the quantity of protein pairs within these combinations that were located in the same subcellular compartment. Immediately following, we further computed the number of protein combinations across age groups (the combinations of protein pairs from different age branches), labeled as Inter_num. Given that the number of protein pairs within an age group is relatively smaller compared with the combinations across age groups (Intra_num < Inter_num), we randomly selected an equal number of protein pairs from the cross-age group combinations that matched the Intra_num in the random selected protein pairs.

By comparing the quantities of these two groups (intra-branch vs. inter-branch), we were able to estimate whether proteins with the same origination branch tend to localize within the same subcellular compartment. To quantify this tendency, we employed an excess percentage metric, which calculates the excessed ratio of the number of co-localized protein pairs within the same age group (intra-branch) relative to the number of co-localized protein pairs across age groups (the combinations of protein pairs from different age branches) (inter-branch). To ensure the robustness and consistency of our comparison results, we repeated this process 100 times. Through multiple iterations of the experiment, we were able to assess the localization tendencies of protein pairs. In this analysis, we used the longest transcript to represent the gene and only retained entries that were localized to a single subcellular compartment.

### The Evaluation of Co-function for Protein Pairs with the Same Origination Branch

We consider a pair of proteins to participate in the same pathway and have a co-functional property if they share at least one identical KEGG pathway ID. To examine whether proteins encoded by genes from the same evolutionary branch are co-function, we first calculate the proportion of proteins on a certain pathway *K*_1_ among all proteins, which is the probability of a protein functioning on that pathway *K*_1_, which we denote this probability as *P*(*K*_1_). Obviously, the probability of two proteins simultaneously located on *K*_1_ under random conditions is calculated by *P*^2^(*K*_1_). Assuming there are *N* pathways, the probability of co-function on all *N* pathways is $\sum_{i=1}^{i=N}P^{2}(K_{i})$. From this, we can calculate the expected number of co-functional proteins in each evolutionary branch (this number was denoted by *E*), and at the same time, we can count the observed number of co-functional protein pairs (this number was denoted by *O*) (see Result section for the number details). Comparing *O* with *E* can determine whether protein pairs encoded by genes from the same evolutionary branch are co-function. When (*O* − *E*)/*E* is greater than 0, it indicates that protein pairs are co-functional.

### The Measurement of Sequence Divergence for Proteins Localized in Different Subcellular Compartments

We used the average pairwise divergence (*D* value) among orthologous regions to measure evolutionary speed. We use a method similar to Julenius and Pedersen (2006) and Korunes and Samuk (2021) to calculate the average pairwise divergence *D*. The protein encoded by the longest transcript in alternative splicing was used as the representative of the gene in *D. melanogaster*, and we used CD-HIT to cluster all the representatives with the parameters “-c 0.5 -n 3” (Fu et al. 2012). The clusters with the number of members less than two were excluded in our following analysis. Meanwhile, the clusters with all the proteins localizing in a single subcellular compartment were retained since multiple subcellular localizations of proteins encoded by genes may impact the reliability of the analysis results. Upon further examination of these preserved clusters and their corresponding subcellular localization data, we will retain subcellular compartments that encompass more than 40 protein clusters in our analysis. All retaining clusters were aligned by MAFFT (Nakamura et al. 2018) at default parameters. Based on the result of sequence alignments, we calculated the *D* value according to the description from Korunes and Samuk (2021).

To exclude the impact of gene age on the analysis of the average pairwise divergence (*D*), we further constrained the clusters to the same or nearly same origination branch. We then examined the relationship between *D* and subcellular localization for protein clusters. In detail, we divided the proteins into four age groups (oldest, older, old, and young), which correspond to br-2, br-1, br0, and br1 to br6. We then calculated the percentage of each originating branch (supplementary fig. S4a, Supplementary Material online) for proteins within their corresponding cluster (generated by CD-HIT with parameters -c 0.5 and -n 3). For each cluster, we have four percentages, each corresponding to one of the four age groups. If the ratio of br-2 and br-1 for a given cluster is 100% (indicating that the proteins corresponding to these genes originate from the two branches, and that no proteins from other branches are present in this cluster), such clusters will be further considered for analysis (supplementary fig. S4a, Supplementary Material online). The proteins in these clusters have consistent or closely related origins, allowing for the analysis of the relationship between *D* and subcellular location while minimizing the influence of evolutionary origin on the *D* value.

### Protein–Protein Interaction Network and the Measurement of Interaction Strength

We downloaded the protein–protein physical interaction (PPI) data of *D. melanogaster* from the STRING database. The PPI data can be available at the STRING link https://stringdb-downloads.org/download/protein.physical.links.v12.0/7227.protein.physical.links.v12.0.txt.gz (Szklarczyk et al. 2023), and we retained interaction pairs with a score greater than 700 (the recommended cutoff for selecting confident interaction pairs by the official website). Subsequently, we associated these protein interaction pairs with their corresponding subcellular localization information. To quantify the interaction strength, we counted the number of links for proteins localized in a specific subcellular compartment along with their corresponding gene numbers. Subsequently, the average degree per gene was calculated, which serves as a measure of the interaction density within the corresponding subcellular compartment.

### The Classification of Gene Duplication and the Evolutionary Pattern of Subcellular Localization

We downloaded the paralogous genes for *D. melanogaster* from the FlyBase database (https://ftp.flybase.net/releases/FB2024_01/precomputed_files/orthologs/dmel_paralogs_fb_2024_01.tsv.gz) and filtered out one-to-one paralogous gene pairs. Based on our gene age data, we classified the genes as parental and offspring genes, where the parental gene originated earlier than the offspring gene within the paralogous gene pairs. We further categorize duplication events into two types, ancient duplication and recent duplication, based on the branch of parental and offspring genes on the evolutionary tree.

1. Ancient duplication: We define duplication events as ancient duplication if the parental gene is located in the br-2 and the offspring gene is located in the br-1 or br0 branch. Additionally, if the parental gene is located in the br-1 and the offspring gene is located in the br0, it is also considered an ancient duplication. These gene pairs are thought to have undergone duplication in earlier periods due to their long-term co-existence.
2. Recent duplication: Except for the ancient duplication events mentioned above, all other duplication events are classified as recent duplication, indicating that the duplication of these gene pairs occurred later.

Additionally, we further defined the patterns of subcellular localization changes between offspring genes and their corresponding parental genes, which can be categorized into five categories: K (keep), SE (shrink and extend), S (shrink), E (extend), and KE (keep and extend) (Fig. 5a). Specifically, K indicates that the offspring gene and parental gene retain exactly the same subcellular localizations; SE indicates that the offspring gene may lose some subcellular localizations relative to the parental gene while acquiring new localizations; S indicates that the offspring gene might lose subcellular localizations without acquiring new localizations; E indicates that the offspring gene has lost all subcellular localizations of the parental gene but has acquired new subcellular locations; and KE indicates that the offspring gene has retained all subcellular localizations relative to the parental gene while also acquiring new subcellular compartments (Fig. 5a). Note that the above classification of subcellular localization patterns is only a relative categorization (with the offspring gene relative to the parental gene) and does not reflect their actual changes in subcellular localization.

### Statistical Analysis

The statistical analyses used in this study, including Mann–Whitney *U* test, Fisher's exact test, Spearman's correlation analysis, and Chi-square test, were performed by SciPy (version 1.10.1) (Virtanen et al. 2020) python package, which is executed in Python platform (version 3.8.17).

## Supplementary Material

msaf038_Supplementary_Data

## Data Availability

All data supporting the findings of this study are available in the online Supplementary Materials.
