# Supplementary material for: Subcellular Enrichment Patterns of New Genes in Drosophila Evolution
Source: Mol Biol Evol. 2025 Feb 7;42(2):msaf038. doi: 10.1093/molbev/msaf038 (PMC11843443; doi:10.1093/molbev/msaf038)
Supplement: msaf038_Supplementary_Data [file msaf038_supplementary_data.zip › 2. Supplementary Materials.pdf]

# Supplementary Materials

**Evolutionary tree in the Newick format used for dating gene age of *D. melanogaster***

(Bactrocera_dorsalis:126.00000000,(Scaptodrosophila_lebanonensis:65.20000000,((Drosophila_grimshawi:42.30000000,((Drosophila_hydei:17.95051275,Drosophila_mojavensis:17.95051275)'14':13.10269011,(Drosophila_novamexicana:4.24616200,Drosophila_virilis:4.24616200)'13':26.80704086)'11':11.24679714)'10':7.80000000,(Drosophila_willistoni:50.10000000,((Drosophila_azteca:7.71938000,(Drosophila_persimilis:3.37033600,Drosophila_pseudoobscura:3.37033600)'19':4.34904400)'9':29.46520000,(Drosophila_ananassae:33.86362600,((Drosophila_yakuba:8.18063000,(Drosophila_erecta:4.98205500,Drosophila_orena:4.98205500)'6':3.19857500)'30':3.26046733,((Drosophila_sechellia:3.31811200,Drosophila_simulans:3.31811200)'22':2.55700891,Drosophila_melanogaster:5.87512091)'8':5.56597642)'29':22.42252867)'27':3.32095400)'35':12.91542000)'43':0.00000000)'42':15.10000000)'40':60.80000000);

**Evolutionary tree in the Newick format used for dating gene age of *D. virilis***

((((((((((Drosophila_virilis:4.24616200,(Drosophila_novamexicana:1.13964000,Drosophila_americana:1.13964000)'13':3.10652200)'11':5.16127400,Drosophila_montana:9.40743600)'10':21.64576686,(Drosophila_hydei:17.95051275,Drosophila_mojavensis:17.95051275)'14':13.10269011)'19':0.82395714,(Drosophila_lacertosa:23.52058500,Drosophila_melanica:23.52058500)'9':8.35657500)'22':8.97726250,(Drosophila_albomicans:1.34332000,Drosophila_nasuta:1.34332000)'8':39.51110250)'6':1.44557750,Drosophila_grimshawi:42.30000000)'30':2.10000000,Drosophila_busckii:44.40000000)'29':5.70000000,(Drosophila_willistoni:50.10000000,(((Drosophila_persimilis:3.37033600,Drosophila_pseudoobscura:3.37033600)'27':4.34904400,Drosophila_azteca:7.71938000)'35':29.46520000,(Drosophila_ananassae:33.86362600,(((Drosophila_sechellia:3.31811200,Drosophila_simulans:3.31811200)'43':2.55700891,Drosophila_melanogaster:5.87512091)'42':5.56597642,(Drosophila_yakuba:8.18063000,(Drosophila_erecta:4.98205500,Drosophila_orena:4.98205500)'40':3.19857500)'48':3.26046733)'51':22.42252867)'47':3.32095400)'39':12.91542000)'56':0.00000000)'55':15.10000000,Scaptodrosophila_lebanonensis:65.20000000)'61':60.80000000,Bactrocera_dorsalis:126.00000000);

**This work provides more accurate annotation of gene age in *D. melanogaster***

To investigate the gene age confidence from this study, we utilized the same pipeline with the current work to re-date the gene age of *D. melanogaster* using 11 reference

genomes, which include several long-read sequencing genomes (Figure 1A in the Supplementary Materials). This will facilitate an easier and unbiased comparison for gene age between this work and GenTree (http://gentree.ioz.ac.cn/download/dm6_ver78_age.tsv), given that GenTree relies on 11 reference species for gene age dating of *D. melanogaster*. For the sake of convenience, we refer to the gene age estimated using the 11 reference genomes as DroAge24 in this study. Please note that the reference genomes used in DroAge24 and GenTree differ, as we include long-read sequencing genomes. We also improved the previous dating method by handling the masked exon regions in focal genomes. In the comparison between DroAge24 and GenTree, we first noticed the gene number in each branch between our result and the result from GenTree showed significant positive correlation ($R^2 = 0.81$, *P-value* = 4.3e-10), implying a general consistency between the two gene age versions in terms of the gene count (Figure 1A in this Supplementary Material and Supplementary Table S4). Second, we further divided our comparisons into three types: genes with the same gene age between GenTree and DroAge24 (marked comparable in Supplementary Table S5), genes having an older age compared with GenTree (marked older in Supplementary Table S5), and genes having a younger age compared with GenTree (marked younger in Supplementary Table S5). For simplification, we abbreviated the three comparisons as same, older and younger. We found that 94.3% genes have the same gene age, also implying the consistency of the two-age versions. However, by introducing the high-quality reference genomes and developing an improved exon handling procedure, we annotated 3.2% younger genes and 2.5% older genes compared with GenTree (Figure 1B in the Supplementary Materials, Figure 2 in the Supplementary Materials, and Supplementary Table S5). Meanwhile, the consistency between the two age versions in both young and old age groups reach to an accepted level. For example, the consistency of genes annotated as young reaches ~80% between DroAge24 and GenTree, while the consistency between those annotated as old genes exceeds 97% (Figure 1C in the Supplementary Materials). Please note that in our GageTracker paper (Fang et al. 2024), we also performed this comparison using reference genomes from different assembly versions (generated by NGS technology).

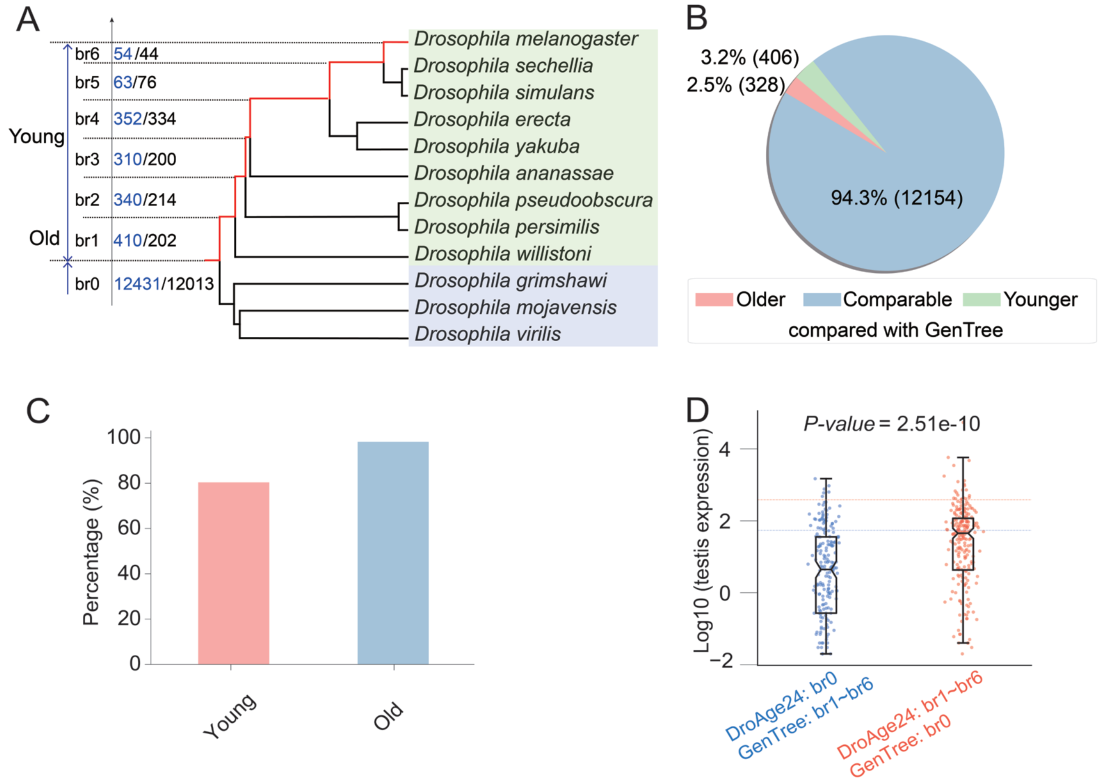


**Figure 1 in the Supplementary Materials. The comparison of gene age between DroAge24 and GenTree.** (A) The evolutionary tree of *Drosophila* is utilized for re-evaluating gene age based on 11 reference species (including the long-read sequencing genomes). The gene age generated by the reference tree and pipeline used in this work is labeled as DroAge24. Blue numbers in branches signify gene counts in DroAge24, while other numbers in black represent gene counts in GenTree. (B) The comparisons are categorized into three types: gene ages in DroAge24 are younger than GenTree (younger), comparable between DroAge24 and GenTree (comparable), or older in DroAge24 compared to GenTree (older). (C) Consistency comparison between DroAge24 and GenTree in two age categories (young and old) is based on considering gene ages as the same if they both fall into the young group (br1~br6) in both GenTree and DroAge24 or fall into the old group (br0) in both GenTree and DroAge24. (D) Testis expression comparison is conducted for two groups of genes: genes' age at br0 in DroAge24 but at br1~br6 in GenTree, and genes' age at br1~br6 in DroAge24 but at br0 in GenTree. The expression data were retrieved from FlyAtlas 2.0.

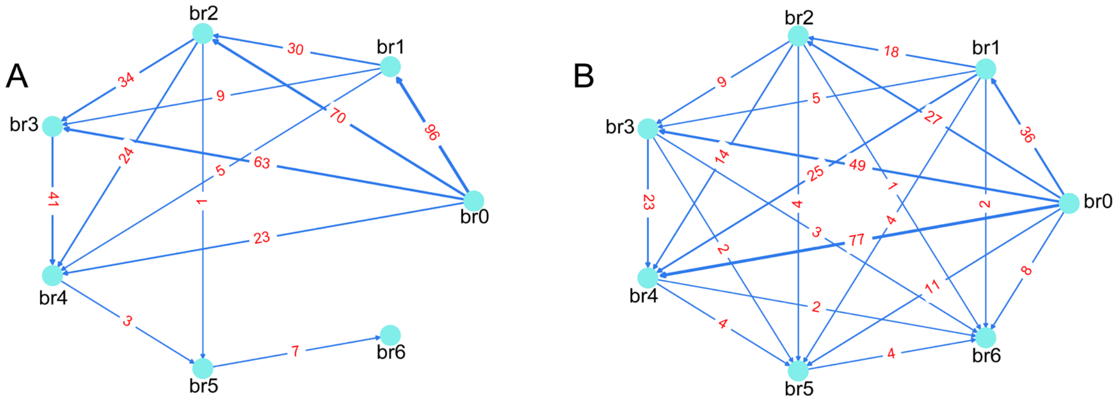

**Figure 2 in the Supplementary Materials**. **The number of genes with different age annotation between DroAge24 and GenTree.** (A) The number of genes that are annotated as a relatively younger age in DroAge24 compared to GenTree. The numbers along the arrow line indicate the gene counts that were annotated as relatively younger in DroAge24 compared to GenTree. For example, a straight line with an arrow points from br2 to br3, and the number 34 is marked on the line, indicating that 34 genes in GenTree have moved from the br2 to the relatively younger br3 in DroAge24. (B) The number of genes that annotated as a relatively older in DroAge24 compared to GenTree. The number next to the arrow line represents the count of genes that were annotated as older in DroAge24 than in GenTree. For example, a straight line with an arrow points from br2 to br3, and the number 9 is marked near the line, indicating that these 9 genes are labeled as br2 in DroAge24, but GenTree considers them as relatively younger genes (br3). The thickness of the line is positively correlated with the number.

To further determine which gene age dating pipeline is more reliable, we investigate the expression feature of the above younger and older genes across the two-age version (DroAge24 vs. GenTree). The older genes typically exhibit broader expression pattern and are often expressed in multiple tissues, which can be measured by $\tau$ index (Yanai et al. 2005). The index ranges from 0 to 1, reflecting the degree of tissue-specific expression for a gene; a high $\tau$ value indicates specific expression across tissues, while a low $\tau$ value signifies broader expression. Using male expression data from FlyAtlas2 (Leader et al. 2018), our results show that the older genes have a significantly smaller mean $\tau$ index compared with younger genes (Mann-Whitney U test, *P-value* = 0.0001103). These analyses implied that the older genes exhibit a broader expression level compared to younger genes according to our annotation. We also categorized genes with different gene ages into two groups according to gene age in DroAge24 and GenTree. One group consists of genes that meet the following criteria: in our study, we assigned these genes to the old branch (br0), while GenTree assigned them to the new (young) branch (br1~br6). The other group includes genes that were classified in our study as belonging to the new (young) branch (br1~br6), but GenTree assigned them to the old gene branch (br0). We downloaded testis expression data from FlyAtlas (version 2) (Leader et al. 2018), and examined the expression of the two gene groups in the *D. melanogaster* male testis. We found that the latter group is more likely to be expressed in the testis than the former (Mann-Whitney U test, *P-value* = 2.51e-10) (Figure 1D in the Supplementary Materials). Together, from the expression analysis, we showed that these genes with different gene ages supported that our result is more reliable compared with GenTree. Additionally, these data also imply that the accuracy of gene age dating can be improved by including long-read sequenced genomes and more advanced dating methodology. Please also note that in our GageTracker paper (Fang et al. 2024), we also performed this comparison using reference genomes from different assembly versions (generated by NGS).

We also utilized homologous data recorded at FlyBase (Drysdale and Consortium 2008; Thurmond et al. 2019), available for download at the following link: FlyBase Homologous Data, https://ftp.flybase.net/releases/FB2022_01/precomputed_files/orthologs/dmel_orthologs_in_drosophila_species_fb_2022_01.tsv.gz. This data allowed us to assess which gene age annotations from GenTree and DroAge24 are more consistent with gene ages determined based on homologous information from FlyBase. To perform this analysis, we first dated the gene ages according to the homologous information recorded in “drosophila_species_fb_2022_01.tsv.gz”. For convenience, we termed the gene age estimated by FlyBase homologs as FlyBaseAge. Subsequently, we conducted comparisons between GenTree and FlyBaseAge, as well as comparisons between DroAge24 and FlyBaseAge. We used the number of differences in evolutionary branches measure the supportiveness of FlyBaseAge to the two gene age types (DroAge24 and GenTree). For example, if a gene is on the place of br2 and br5 in GageTracker and FlyBaseAge, respectively, the difference in two age version is 3 (the absolute value of 5 minus 2). In the discrepancy gene age between GenTree and DroAge24, we found that DroAge24 has 352 genes that have closed gene age with FlyBaseAge, however GenTree have 319 genes that have closed branch assignment with FlyBaseAge (Supplementary Table S5). This analysis supports that gene ages estimated by FlyBaseAge are more aligned with DroAge24 rather than GenTree (DroAge24 vs. GenTree). Please note that in our GageTracker work, we used 11 other reference genomes (different assembly versions compared with the current work, and our current work employed several long-read sequencing genomes) and three types of orthologous data to comprehensively assess the accuracy of age annotation pipeline (Fang et al. 2024).

**The ctl file and command lines for dating the gene age of *D. melanogaster* using GageTracker are provided bellow**.

```
# write the branch name from young to old
# The ctl file is named dm.ctl, and its contents are shown in the table below
branch = ["br6", "br5", "br4", "br3", "br2", "br1", "br0", "br-1", "br-2"]
old_br = ["br0", "br-1", "br-2"]
outpath = "/data/chuand/dm_subcellular/data/dm_age"
target = "/data/chuand/dm_subcellular/data/genome/dmelanogaster.fasta"
annotation = "/data/chuand/dm_subcellular/data/Drosophila_melanogaster.BDGP6.32.57.gtf" #
version 110

# DO NOT include the branch belonging to our target species
reference["br-2"] = ["/data/chuand/dm_subcellular/data/genome/bdorsalis.fasta"]
```

```
reference["br-1"] = ["/data/chuand/dm_subcellular/data/genome/slebanonensis.fasta"]
reference["br0"] =
["/data/chuand/dm_subcellular/data/genome/dvirilis.fasta","/data/chuand/dm_subcellular/data/g
enome/dnovamexicana.fasta","/data/chuand/dm_subcellular/data/genome/dmojavensis.fasta","/
data/chuand/dm_subcellular/data/genome/dhydei.fasta","/data/chuand/dm_subcellular/data/gen
ome/dgrimshawi.fasta"]
reference["br1"] = ["/data/chuand/dm_subcellular/data/genome/dwillistoni.fasta"]
reference["br2"] = ["/data/chuand/dm_subcellular/data/genome/dpseudoobscura.fasta",
"/data/chuand/dm_subcellular/data/genome/dpersimilis.fasta",
"/data/chuand/dm_subcellular/data/genome/dazteca.fasta"]
reference["br3"] = ["/data/chuand/dm_subcellular/data/genome/dananassae.fasta"]
reference["br4"] = ["/data/chuand/dm_subcellular/data/genome/dorena.fasta",
"/data/chuand/dm_subcellular/data/genome/derecta.fasta",
"/data/chuand/dm_subcellular/data/genome/dyakuba.fasta"]
reference["br5"] = ["/data/chuand/dm_subcellular/data/genome/dsimulans.fasta",
"/data/chuand/dm_subcellular/data/genome/dsechellia.fasta"]
age = " dm_age"
voting = 0.5
```

The following command lines can help us to date the gene age of *D. melanogaster* and extract the age list of protein-coding genes.

```
nohup GageTracker dm.ctl -p 10 --tantannomsk
GageTracker_r1.0/gage_diff dm.ctl
```

## Reference


Drysdale R, Consortium F. 2008. FlyBase: a database for the Drosophila research community. Methods Mol Biol 420:45-59.

Fang C, Dong C, Wang C, Xiong F, Lu S, Fang W, Li T, Gan X, Yang L, Zeng H. 2024. GageTracker: a tool for dating gene age by micro-and macro-synteny with high speed and accuracy. BioRxiv:2024.2008. 2028.610050.

Leader DP, Krause SA, Pandit A, Davies SA, Dow JAT. 2018. FlyAtlas 2: a new version of the Drosophila melanogaster expression atlas with RNA-Seq, miRNA-Seq and sex-specific data. Nucleic Acids Res 46:D809-D815.

Thurmond J, Goodman JL, Strelets VB, Attrill H, Gramates LS, Marygold SJ, Matthews BB, Millburn G, Antonazzo G, Trovisco V. 2019. FlyBase 2.0: the next generation. Nucleic Acids Res 47:D759-D765. Drysdale R, Consortium F. 2008. FlyBase: a database for the Drosophila research community. Methods Mol Biol 420:45-59.

Yanai I, Benjamin H, Shmoish M, Chalifa-Caspi V, Shklar M, Ophir R, Bar-Even A, Horn-Saban S, Safran M, Domany E, et al. 2005. Genome-wide midrange transcription profiles reveal expression level relationships in human tissue specification. Bioinformatics 21:650-659.
