# Supplementary material for: Subcellular Enrichment Patterns of New Genes in Drosophila Evolution
Source: Mol Biol Evol. 2025 Feb 7;42(2):msaf038. doi: 10.1093/molbev/msaf038 (PMC11843443; doi:10.1093/molbev/msaf038)
Supplement: msaf038_Supplementary_Data [file msaf038_supplementary_data.zip › 3._Supplementary_Figures.pdf]

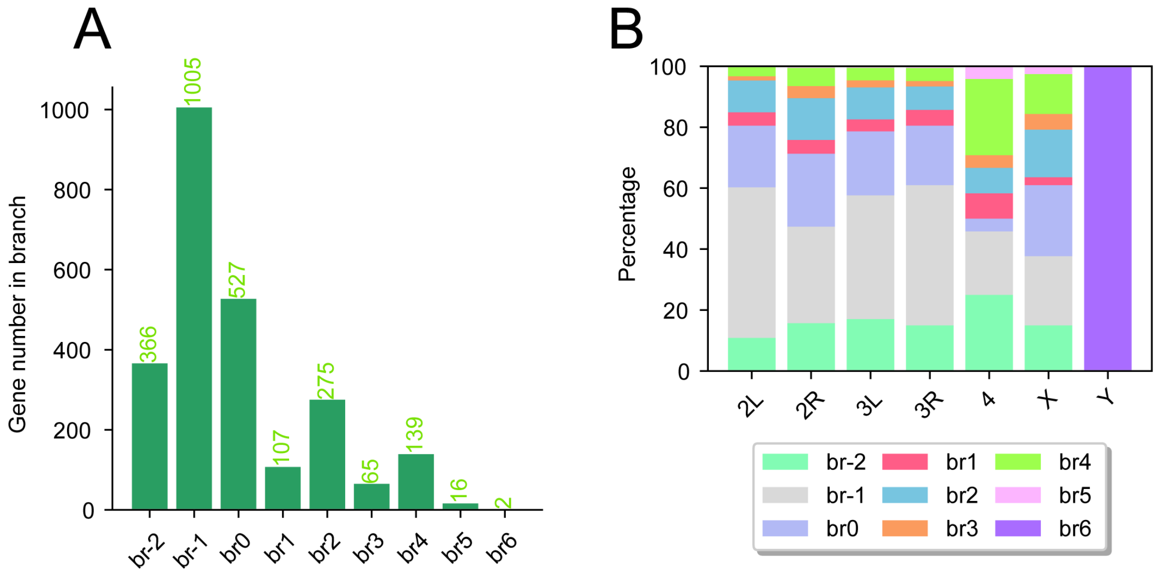

Supplementary Figure S1. The distribution of non-coding RNA on branches and chromosomes. (A) The distribution of non-coding RNA across branches, where we designate br-2 as the oldest branch, br-1 as the older branch, br0 as the old branch, and br1~br6 as the young branches. (B) The gene age distribution of non-coding RNA on different chromosomes.

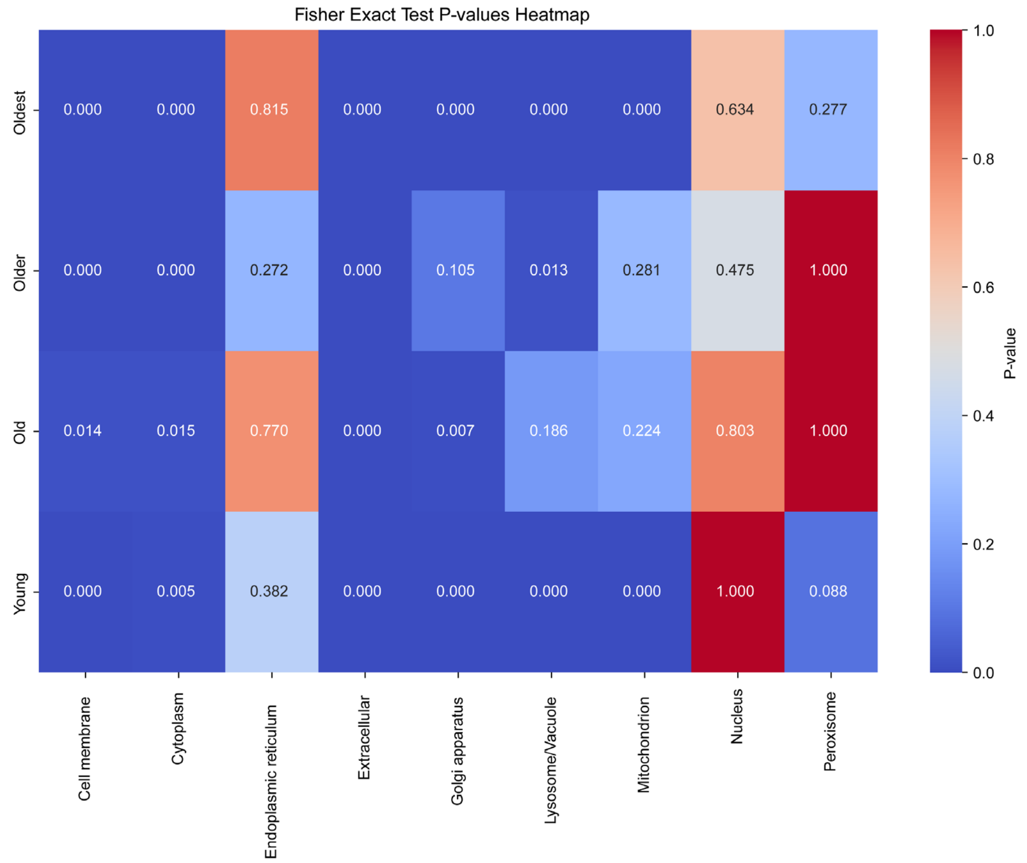

Supplementary Figure S2. The *P-values* of Fisher exact test. We analyzed the counts of proteins encoded by genes from different origin branches in subcellular localization and calculated the expected counts under random localization. Fisher exact tests were performed for proteins in each origin branch and each subcellular compartment.

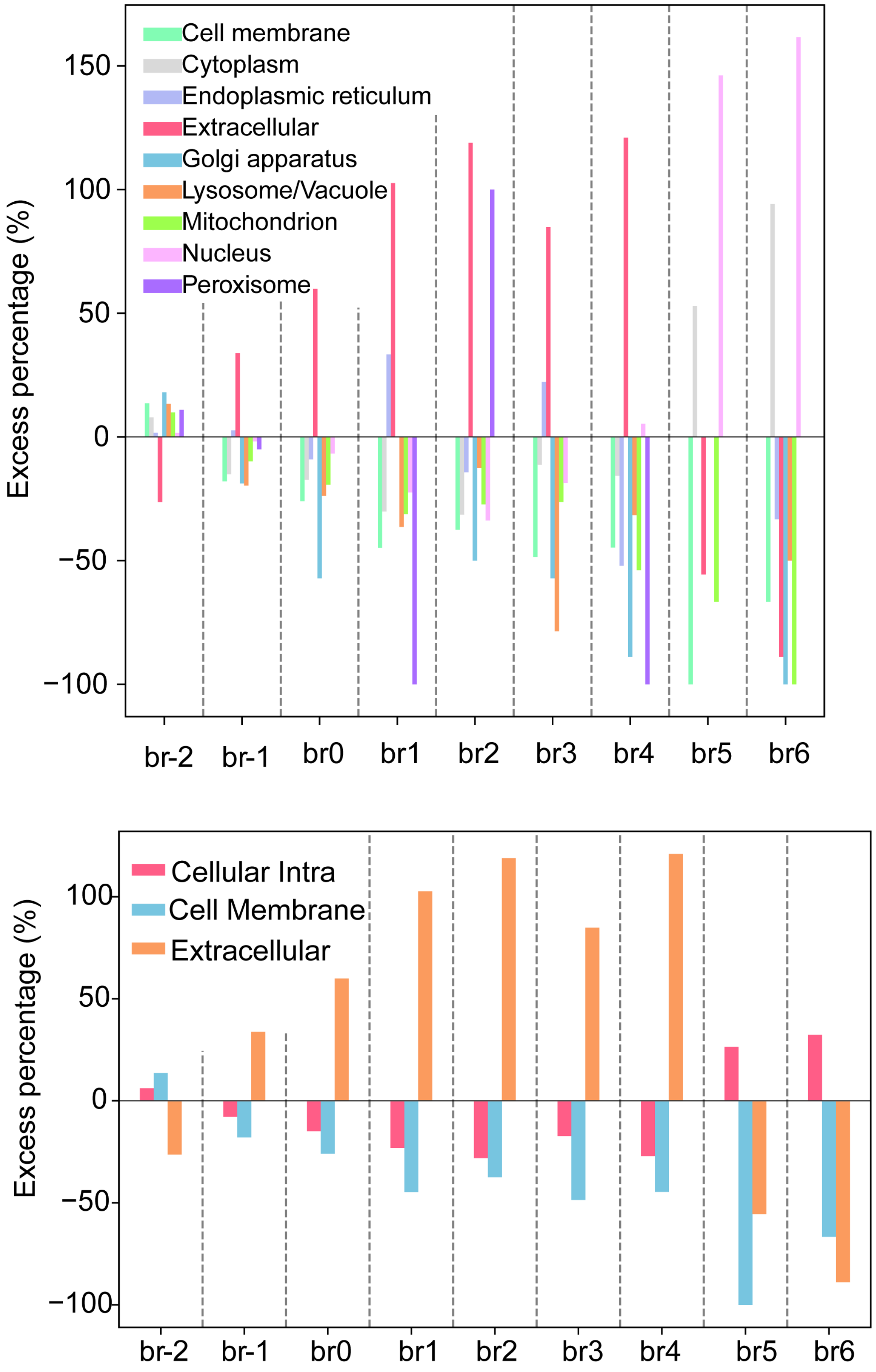

Supplementary Figure S3. Subcellular localization enrichment analysis. (A) The enrichment analysis of proteins in 9 subcellular compartments for the 9 age branches. (B) The enrichment analysis in 3 broader subcellular compartments including intracellular, cell membrane and extracellular space.

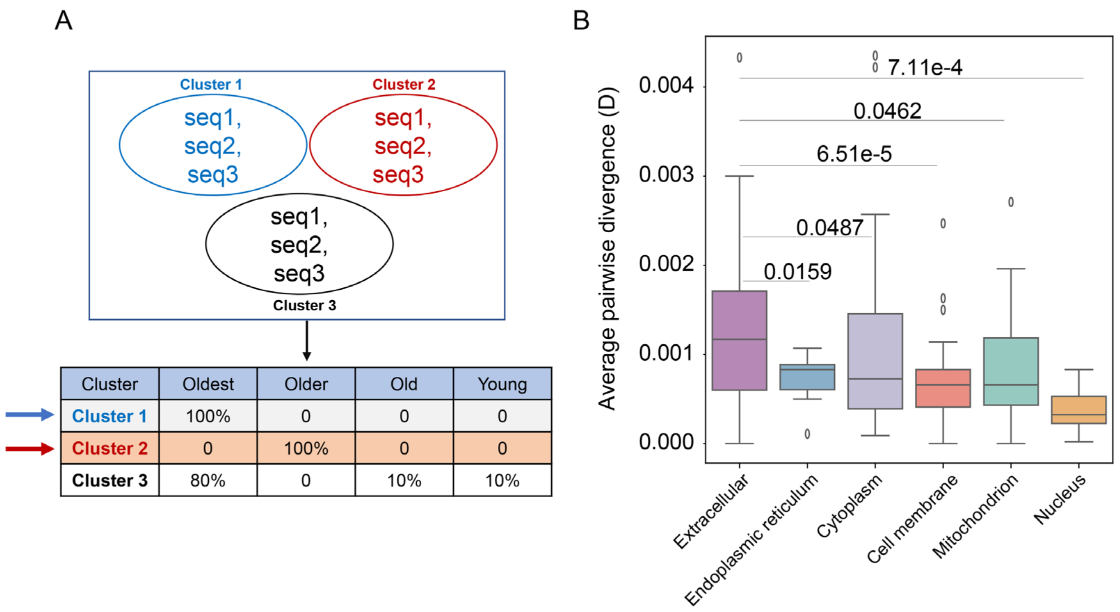

Supplementary Figure S4. (A) This panel illustrates how we eliminate the influence of origin age on the analysis of evolutionary rates of proteins across different subcellular compartments. (B) This panel displays the average pairwise divergence of age-restricted clusters across different subcellular compartments. The age-constrained protein clusters in the extracellular space exhibit a significantly faster evolutionary rate.

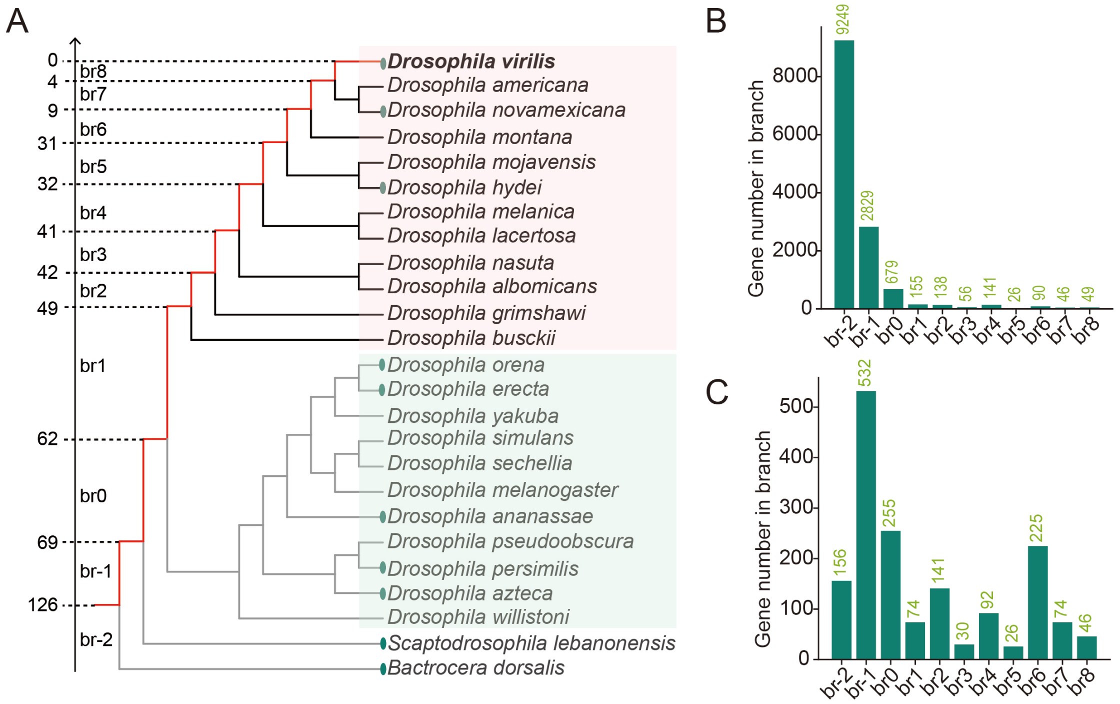

Supplementary Figure S5. Gene age dating of *D. virilis*. (A) The evolutionary tree for dating gene age of *D. virilis*. Species in light red background are members of the focal subgenus *Drosophila*, and the red line indicates the divergence direction of our focal species *D. virilis*, and those species in light green background are members of the *Sophophora* subgenus. (B) Age distribution of protein-coding genes in *D. virilis*; (C) Age distribution of lncRNA genes in *D. virilis*.

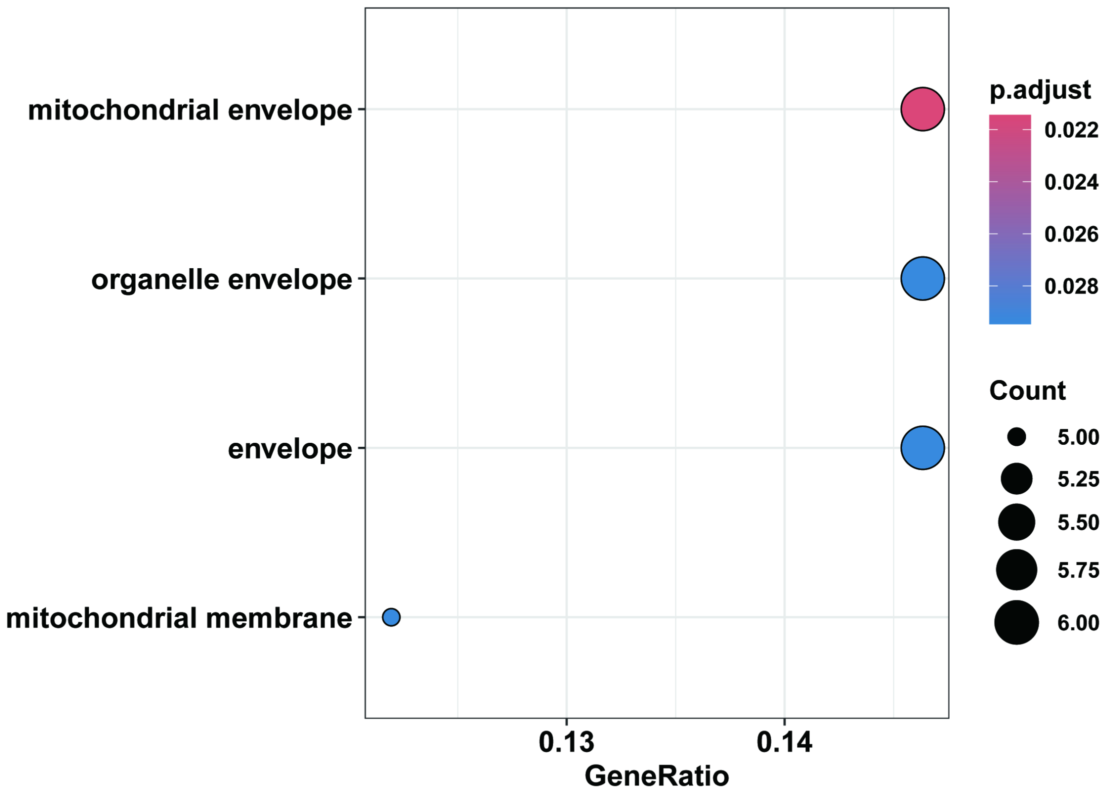

Supplementary Figure S6. The enrichment analysis for young proteins that localize in mitochondrion.
